# 3D printed Bouncy Bioglass enhances articular cartilage regeneration

**DOI:** 10.1016/j.bioactmat.2026.09.023

**Published:** 2026-09-22

**Authors:** Agathe Heyraud, Francesca Tallia, Talal Al-Jabri, Arianna De Mori, Qingyue E. Shi, Diogo M.M. dos Santos, Jeffrey N. Clark, Chinmay Gupte, Jonathan R.T. Jeffers, Eduardo H.M. Nunes, Gordon Blunn, Justin Cobb, Julian R. Jones

**Affiliations:** a Department of Materials, Imperial College London, London, UK; b Department of Surgery and Cancer, Imperial College London, London, UK; c School of Medicine, Pharmacy and Biomedical Science, University of Portsmouth, Portsmouth, UK; d Universidade Federal de Minas Gerais, Departamento de Engenharia Metalúrgica e de Materiais (DEMET), Belo Horizonte, Pampulha, Brazil; e Department of Mechanical Engineering, Imperial College London, London, UK; f Department of Trauma and Orthopaedics, Imperial College Healthcare NHS Trust, London, UK

**Keywords:** Chondral defect, Hybrids, Sol-gel, Articular cartilage regeneration, 3D printing

## Abstract

Articular cartilage is an avascular tissue with limited intrinsic repair capacity, and untreated defects will deteriorate progressively over time. Lining the ends of bones, it plays a critical role in joint lubrication and shock absorption. Current surgical interventions, such as microfracture, typically result in the formation of mechanically inferior fibrocartilage. There remains a significant unmet clinical need for biomaterials that can support the regeneration of functional articular cartilage. We present a non-cytotoxic sol-gel hybrid scaffold, of composition silica/poly(tetrahydrofuran)/polycaprolactone (SiO_2_/PTHF/PCL-diCOOH), termed ‘Bouncy Bioglass’ to address that need. Our ovine model revealed that Bouncy Bioglass scaffolds with 250 μm channels stimulated hyaline cartilage regeneration. They outperformed scaffolds with 500 μm channel and microfracture alone, by improved integration and filling of the defect site with articular-like cartilage tissue. This tissue showed a significant increase in collagen type II and other primary components of healthy articular cartilage, compared to the tissue formed from the other two conditions. Our hybrid biomaterial, Bouncy Bioglass, enhances the regeneration of articular cartilage and may offer an ‘off-the-shelf’ synthetic implant option to treat localised articular defects and prevent their continued degeneration.

## Highlights

• The first scaffold material that promotes articular cartilage regeneration *in vivo*.
• Scaffold architecture and surface chemistry are key to the implant's success.
• Our 3D printed “Bouncy Bioglass” scaffold with 250 μm channel size was key.
• Good tissue integration, hyaline-like cartilage architecture, no bone cysts.
• No systemic cytotoxicity from the implants in rat models.

## Graphical abstract

**Figure:**
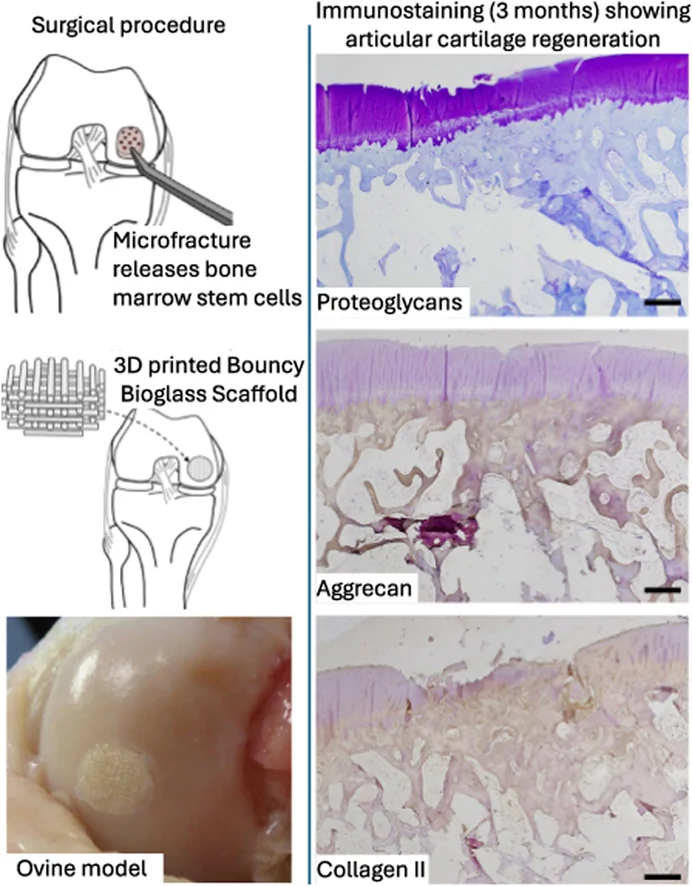

## 1 Introduction

Articular cartilage is a unique material able to facilitate motion, distribute load and absorb shock [1], but unlike bone, it is avascular and has limited capacity for self-repair following injury [2]. Localised articular cartilage defects, commonly caused by acute trauma or repetitive mechanical stress, are present in two thirds of patients suffering from knee pain and can significantly impair joint function [3]. Without effective intervention, such defects can progress and compromise the surrounding cartilage and lead to long-term morbidity [4]. Among current surgical strategies, microfracture, a marrow-stimulation technique (Fig. 1a), is widely used for small chondral defects (<2 cm^2^). However, it forms fibrocartilage, rich in collagen type I [5], which is less resilient than articular cartilage. While short term improvements are seen, durability beyond 5 years is poor, often resulting in treatment failure and additional surgery [6]. Another current treatment option is autologous chondrocyte implantation (ACI, Fig. 1b), a 2-step surgical procedure [6]. Healthy articular cartilage is harvested arthroscopically from a low load-bearing region of the affected knee and the isolated chondrocytes are expanded for 14 to 21 days to then be injected into the defect under a periosteal flap (PF) [7]. Despite theoretical advantages over microfracture, ACI is associated with extended recovery time, increased surgical lead time, and only marginal improvements [8]. Another alternative is mosaicplasty, a one-step procedure (Fig. 1c), in which osteochondral plugs are transplanted from a low weight-bearing region to the defect site [9]. Although this technique can address larger defects and avoids the need for cell expansion, it also results in fibrocartilage between the plugs and can cause donor site morbidity and pain [10]. While mosaicplasty reduces the risk of infections and rejection, its clinical benefits are still comparable to simpler approaches. These surgical techniques all lead to fibrocartilage, risks of further degradation and high rates of surgical re-intervention, highlighting the need for more effective regenerative options.

**Fig. 1:**
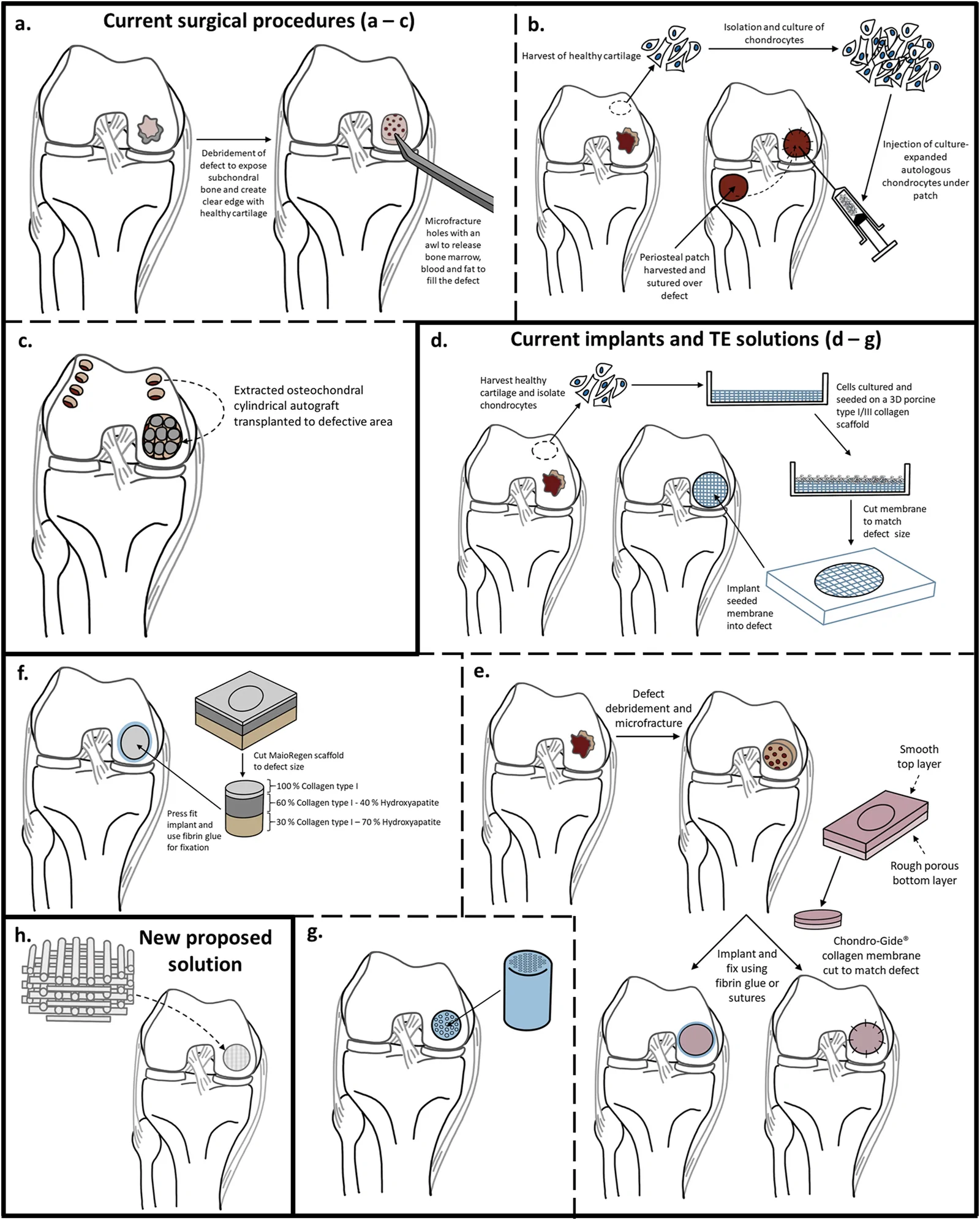
Schematics of current surgical procedures for articular cartilage regeneration: a. microfracture, b. Autologous Chondrocyte Implantation (ACI), c. Mosaicplasty, osteochondral autografts, d. Matrix-induced Autologous Chondrocyte Implantation (MACI), e. Autologous Matrix-induced Chondrogenesis (AMIC® Chondro-Gide®), f. MaioRegen™, g. CARTIHEAL AGILI-C™, and h. the new proposed Bouncy Bioglass solution SiO_2_/PTHF/PCL-diCOOH sol-gel hybrid scaffold implant.

Biomaterials have been introduced in the early 2000s to improve efficacy of microfracture and ACI [11]. One example is MACI®, Matrix-induced Autologous Chondrocyte Implantation (Fig. 1d), a two-step procedure to treat articular cartilage lesion sizes ranging between 2 and 10 cm^2^. First, healthy cartilage is biopsied, and chondrocytes are isolated and cultured onto a collagen membrane [12]. However, during expansion on two-dimensional membranes, a major risk is that cells may lose their chondrocyte phenotype[13] and not form the correct tissue. This explains the issue raised that after > 1 year, biopsies revealed unorganised cells in half the specimens, surrounded by fibrocartilage or fibrous matrix [14]. A second example is AMIC®, Autologous matrix-induced chondrogenesis (Geistlich Pharma, Switzerland), which combines microfracture with matrix-based methods (Fig. 1e), using a collagen membrane, often Chondro-Gide® [15]. Unlike MACI, it is a one-step procedure that does not require harvest of cartilage, avoiding costly and lengthy cell expansion [16]. AMIC outperforms microfracture alone and expands the size of cartilage defects that can be treated by microfracture beyond 2 cm^2^. However, no information on the type of collagen produced was published [17]. Alternatives to the Chondro-Gide membrane used in AMIC include Chondrotissue® (BioTissue, Zurich, Switzerland) and Hyalofast® (Fidia Advanced Biopolymers, Padua, Italy) [18]. Chondrotissue comprises of woven polyglycolic acid fibres impregnated with hyaluronic acid [18] and has demonstrated improved histological outcomes over microfracture alone in a 6-month ovine study [19]. Specifically, the repaired tissue showed evidence of collagen type II and proteoglycan rich matrix, both markers of articular cartilage [19], however, the tissue thickness does not appear fully restored. Another option is Hyalofast, a non-woven porous 3D structure made from semisynthetic derivative of hyaluronic acid with fibres of 10 – 15 μm, inducing mesenchymal stem cells (MSCs) from the bone marrow to differentiate along the chondrogenic lineage [18]. Using Hyalofast for AMIC is often combined with an additional step of extracting bone marrow derived MSCs and platelet-rich plasma (PRP) [20], further complicating the surgical process. Histology analyses of repaired tissue showed some hypocellularity and the presence of fibrous matrix alongside collagen type II [21], suggesting inconclusive regeneration of articular-like tissue. Although AMIC with Chondro-Gide, Chondrotissue or Hyalofast expand the capabilities of microfracture, such as the size of defect tackled, the disadvantages of microfracture are still present with this technique.

Another surgical approach to tackle chondral defects is the use of osteochondral implants that anchor into the underlying bone. However, these require drilling into healthy bone and may cause further degradation of surrounding tissue. Examples of current commercially available osteochondral implants include MaioRegen™ (Finceramica, Faenza S.p.A., Italy) [22] and CARTIHEAL AGILI-C™ (Smith & Nephew, Watford, England) [23] (Fig. 1f and g). MaioRegen can be shaped in surgery but has low strength, whereas AGILI-C is a stiff ceramic scaffold countersunk into the underlying bone. Neither of these mechanical properties match the cartilage or bone host tissues. A recent *in vivo* sheep study on MaioRegen osteochondral implant to treat knee defects showed improvement of bone regeneration when compared to microfracture. However, it also demonstrated that the superficial layer of the defect treated with MaioRegen was composed of predominantly fibrous tissue transitioning toward fibrocartilage after 12 months, confirming the inability of the biomaterial to support the regeneration of healthy articular cartilage [24]. Agili-C has been extensively evaluated in both in *in vivo* large animal studies (e.g. up to 12-month follow-up study in a goat model vs. microfracture [23]) and clinical trials. Despite some findings suggesting the ability to support the formation of hyaline cartilage, the clinical trials did not include a control group and the follow-ups were limited to short-medium term, with the long-term efficacy yet to be determined. In addition to the fact that it requires the removal of a deep plug of healthy subchondral bone to treat just chondral defects, the surgery is limited to the cases where the margins of the defects are covered with healthy and stable articular cartilage, otherwise a high rate of failure has been observed [25].

In summary, the currently available two-step tissue engineered methods are complex, expensive, and surgery lead times can be long. One-step biomaterial implants introduced in the early 2000s have not shown conclusive evidence of long-term articular cartilage formation. Thus, there is a need for a novel one step ‘off-the-shelf’ technology that causes minimal tissue and subchondral bone trauma and regenerates high-quality articular cartilage instead of fibrocartilage.

Sol-gel hybrids, of composition silica/polytetrahydrofuran/polycaprolactone (SiO_2_/PTHF/PCL-diCOOH) established by Tallia et al. [26], show unprecedented mechanical properties and have demonstrated the ability to withstand cyclic loading, hence the term ‘Bouncy Bioglass’. Another advantage of Bouncy Bioglass is that 3D printable inks can be manufactured to create regular structures. Scaffolds printed with ∼150 μm strut thickness and pore channel size of ∼250 μm (Fig. 1h) exhibited mean failure stress of 1.2 ± 0.2 MPa at 36.0 ± 7.8 % strain [26] matching the lower range of normal physiological activities [27]. These scaffolds exhibited a non-liner elastomeric behaviour, with a stiffening of the sample with increasing strain but the ability to completely recover the deformation up to at least 20 % of their initial height [26], mimicking the behaviour of native cartilage. Increasing the pore channel size to ∼500 μm affected the mechanical properties, with a reduction of mean failure stress to 0.36 ± 0.14 MPa at 31.3 ± 9.5 % strain [28]. When we cultured human bone marrow stem cells (hBMSCs) inside scaffolds with pore channels of 250 μm (42.4 ± 3.9 % porosity), the environment facilitated cell-cell interactions, supporting hBMSCs *in vitro* chondrogenic differentiation and the formation of collagen type II matrix, characteristic of hyaline cartilage [29]. A hypoxic environment was found to further enhance articular cartilage-specific extracellular matrix deposition within these scaffolds, even without chondrogenic supplements [30]. When the cells were cultured in smaller pore channels there was no space for cell-cell interactions and this prevented tissue formation with visible voids in matrix formation [29]. When the channels were larger, the cells flattened against the pore walls and produced a fibrocartilage matrix [29]. Before carrying out *in vivo* studies it was confirmed that ovine bone marrow cells performed in a similar manner to the hBMSCs [31].

The purpose of this study was to investigate the efficacy of our Bouncy Bioglass scaffold for cartilage regeneration *in vivo,* comparing our 3D printed hybrid scaffolds with 250 μm channel size (SC-250) to microfracture and hybrid scaffolds with 500 μm channel size (SC-500). This work was based on the *in vitro* observation that the combination of the material (Bouncy Bioglass) and pore architecture (grid-like structures with 250 μm channel size) provides scaffolds with ability to regenerate articular cartilage, which has never been observed before in one-step biomaterial implant for cartilage regeneration.

## 2 Results and discussion

### 2.1 *In vitro* biodegradation study

Ahead of *in vivo* testing, the dissolution or biodegradation of the scaffolds was assessed in phosphate-buffered saline solution (PBS), testing the mechanical properties after immersion of the scaffolds over six months. SC-250 and SC-500 scaffolds were soaked for six months and samples were analysed at 0-1-3-7 days and 1-3-6 months timepoints. Results of variations in the inorganic/organic (I/O) ratio, chemical composition, morphology and mechanical properties are reported in the supplementary information (Figs. S1–S7, Tables S1–S3).

The degradation of inorganic and organic components of the Bouncy Bioglass scaffolds (SC-250 and SC-500) was evaluated to investigate the effect of dissolution on: i) the channel size and surface features; ii) mechanical properties. Early stage (i.e., the first week) and later stage (1 month, 6 months, and 3 months for ICP-OES analysis solely) dissolution were examined. Consistent with the hydrophobic nature of the organic components of the hybrid material, no swelling was observed during the degradation process. From the ICP results on Si release (Fig. S1), the effect of the channel size is evident, since larger channels (SC-500) led to faster dissolution of Si. This is consistent with the larger surface area per unit mass exposed to the PBS environment, as open channels of 500 μm scaffolds providing more space for PBS solution to flow and exchange through the sample. At early stages (1, 3 and 7 days), samples released Si faster than in later stages. The trend of releasing Si from scaffolds did not appear to plateau within the 6 months and the dissolution rate decreased slowly after the first month, so the dissolution of inorganic portion in scaffold samples is expected to continue for longer. For the organic part, results from ^1^H NMR (Fig. S3) and FTIR (Fig. S2) suggest that the degradation of polymer happened mostly in the first month and it was mostly due to PCL, while it slowed down in the following months but still progressed. FTIR results also confirmed that the architecture of hybrid samples (SC-250 and SC-500 compared in Fig. S2a) does not affect the types of dissolution products from the polymer. The inorganic/organic composition of the SC-250 and SC-500, measured *via* TGA (Table S1), remained at approximately 73-75 wt% organic before and after soaking, with variations <2 %. The differences in the I/O ratios upon soaking are deemed not significant (less than 2 %), confirming the congruent degradation of the inorganic and organic parts upon soaking.

Mechanical behaviour of SC-250 scaffolds was not drastically affected by the soaking in PBS, with the scaffolds showing consistent values of stress and strain at failure and storage modulus before and after 1 and 6 months in PBS (Figures S5-S6-S7, Tables S2–S3). This is consistent with the SEM morphological observation (Fig. S4) that proved that the degradation had only affected the surface morphology without impacting the structure and architecture of the scaffolds, up to 6 months. These outcomes demonstrated that SC-250 scaffolds maintained their mechanical properties upon soaking in PBS for 6 months, suggesting the potential to provide continuous mechanical support while new cartilage tissue is undergoing regeneration *in vivo*, where the degradation is expected to be faster due to the more aggressive environment (both biologically and mechanically) but counteracted by the growth of new tissue within the scaffold channels.

### 2.2 Cytotoxicity testing in rat models

To verify our Bouncy Bioglass material had no systemic cytotoxic effect, the SC-250 implants and control ultra-high molecular weight polyethylene (UHMWPE) discs were implanted subcutaneously in rats for 6 weeks and the organs were analysed for signs of cytotoxicity. No signs of toxicity or morbidity were observed or recorded in any rats; there was no post implantation mortality, no significant changes in body weight, and the weight of the organs upon collection was within the normal range for the nine repeats of each organ. No change in the food or water consumption of each group was observed. Histological evaluation of the Haematoxylin & Eosin (H&E) staining showed no detectable cytotoxicity or morphology changes to the analysed organs [32] (Fig. 2). All specific characteristics of each organ were then evaluated (Fig. 2). The hearts showed normal histoarchitecture of the cardiomyocytes with well-organised and branched cardiac myofibres and cardiomyocyte nuclei [33]. The kidneys showed no morphological changes with a normal appearance of the glomerular architecture, no interstitial and intraglomerular congestion or tubular atrophies. The structure clearly showed the normal architecture with the papilla, inner medulla, inner stripes of outer medulla (ISOM), and outer striped of outer medulla (OSOM) [34]. The livers had normal cellular architecture and binucleation with no distortions. And there were no signs of injury, necrosis, congestion, fatty acid, or haemorrhagic regions around the central vein (CV). The classic hepatic lobule showed portal triad (PTs) at the periphery of the lobule and CV in the centre of the lobule, with hepatocytes arranged in radiating cords from the CV and sinusoidal spaces between the cords [34]. The lungs showed the normal pulmonary architecture consisting of alveolar ducts, sacs, and alveoli [35]. The alveoli are lined by alveolar epithelium. The alveoli were separated from each other by thin inter-alveolar septa, surrounded by a smooth muscle layer and bronchial arteriole [35]. The spleen showed the normal architecture, with white and red pulp. The white pulp is a cylindrical mass of lymphocytes with a central artery (CA) that constitutes the periarterial lymphatic sheath (PALS). Red pulp consists of splenic sinuses surrounded by splenic cords [36]. Given these results showing the lack of systemic toxicity from Bouncy Bioglass SC-250 implants in rat models, these were subsequently implanted in an ovine model to evaluate their ability to regenerate articular cartilage. The *in vivo* study demonstrated the response of the surrounding tissue to the implants, indicating that the material is biocompatible and does not elicit local cytotoxic effects. However, prior to clinical translation, a comprehensive biosafety evaluation in accordance with ISO standards using GLP would be required, including longitudinal blood sampling at predefined time points over a period exceeding six months.

**Fig. 2:**
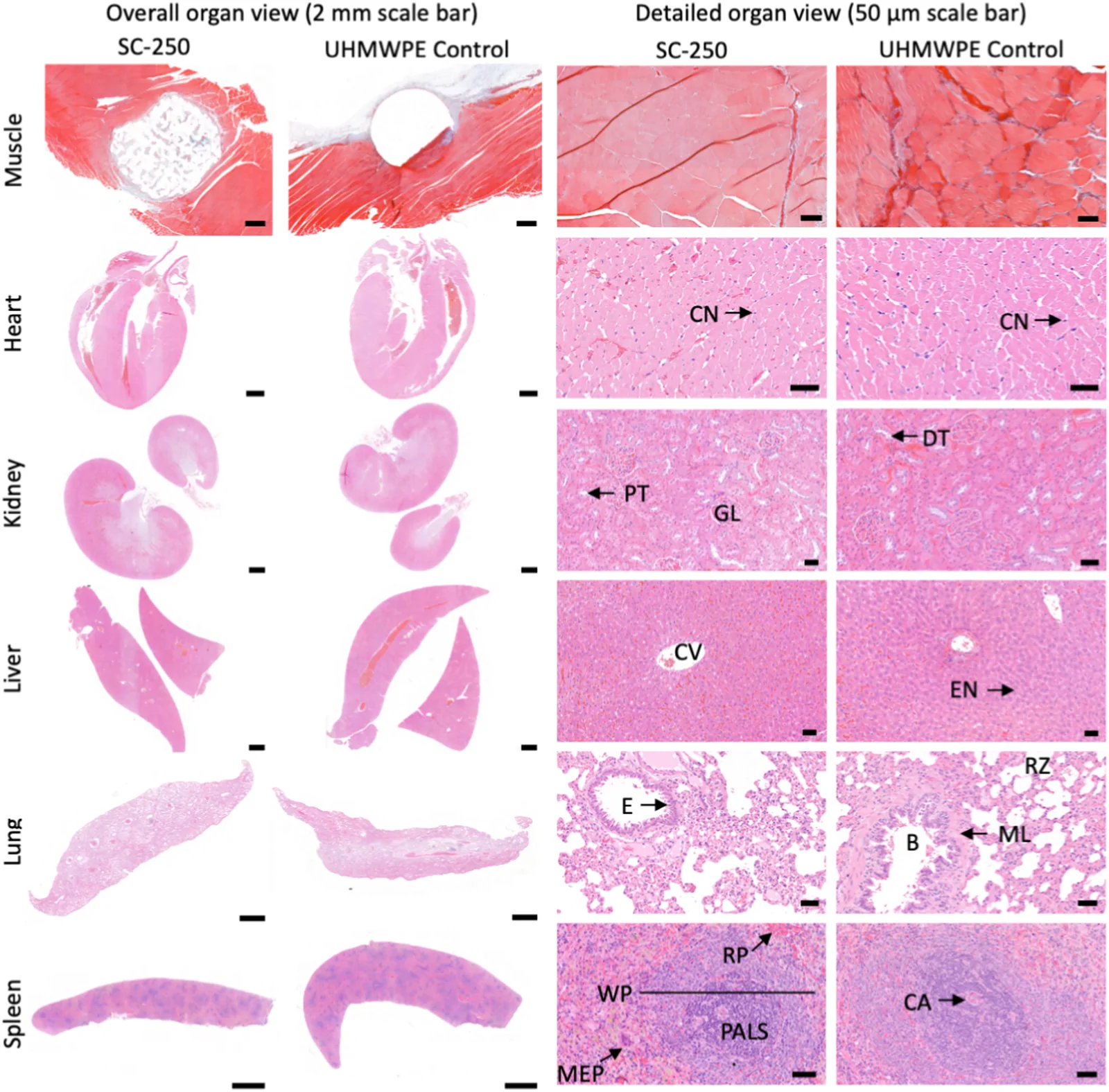
Histology images a subcutaneous implantation of the hybrid implant and UHMWPE control disc in the rat model for 6 weeks. The muscles where the implant/disc was embedded were stained with Masson Trichrome, and the organs with H&E: longitudinal cuts of heart, lung, and spleen; longitudinal and cross section cuts of kidney and liver. Normal morphology was observed for all organs: heart, CN – cardiomyocyte nuclei; kidney, GL – glomerulus, DT – distal tubule, PT – proximal tubule; liver, CV – central vein, EN – endothelial cell; lung, B – bronchiole, RZ – respiratory zone, ML – muscular layer, E − epithelium; and spleen, CA – central artery, PALS- periarterial lymphatic sheath, RP – red pulp, WP – white pulp, MEP – megakaryocytic precursors. A representative rat was used for both conditions. Scale bars of overall organ images 2 mm and detailed view 50 μm.

### 2.3 Qualitative cartilage regeneration in ovine model

An ovine model was used to assess the regenerative capabilities of the SC-250 and SC-500 implants compared to microfracture. After surgery, all sheep were found to have a normal gait and behaviour. After 3 months *in vivo*, the 5 mm diameter defect sites were inspected on the day of extraction and the explants were then visualised histologically using H&E (Fig. 3 SC-250: a, b; SC-500: c, d; microfracture: e, f). The histology images show a cross section of the entire defect, and no scaffold residues were visible, indicating full biodegradation (Fig. 3). Biodegradation occurred faster than in the *in vitro* experiment in PBS. *In vivo,* there is a constant and continuous exchange of fluids, to ensure that the levels of ions and proteins contained in the extracellular matrix are regulated and balanced. This, in combination with potential contribution of enzymatic action and effect of the continuous mechanical stress to which the knee joint is subjected, led to faster degradation kinetics.

**Fig. 3:**
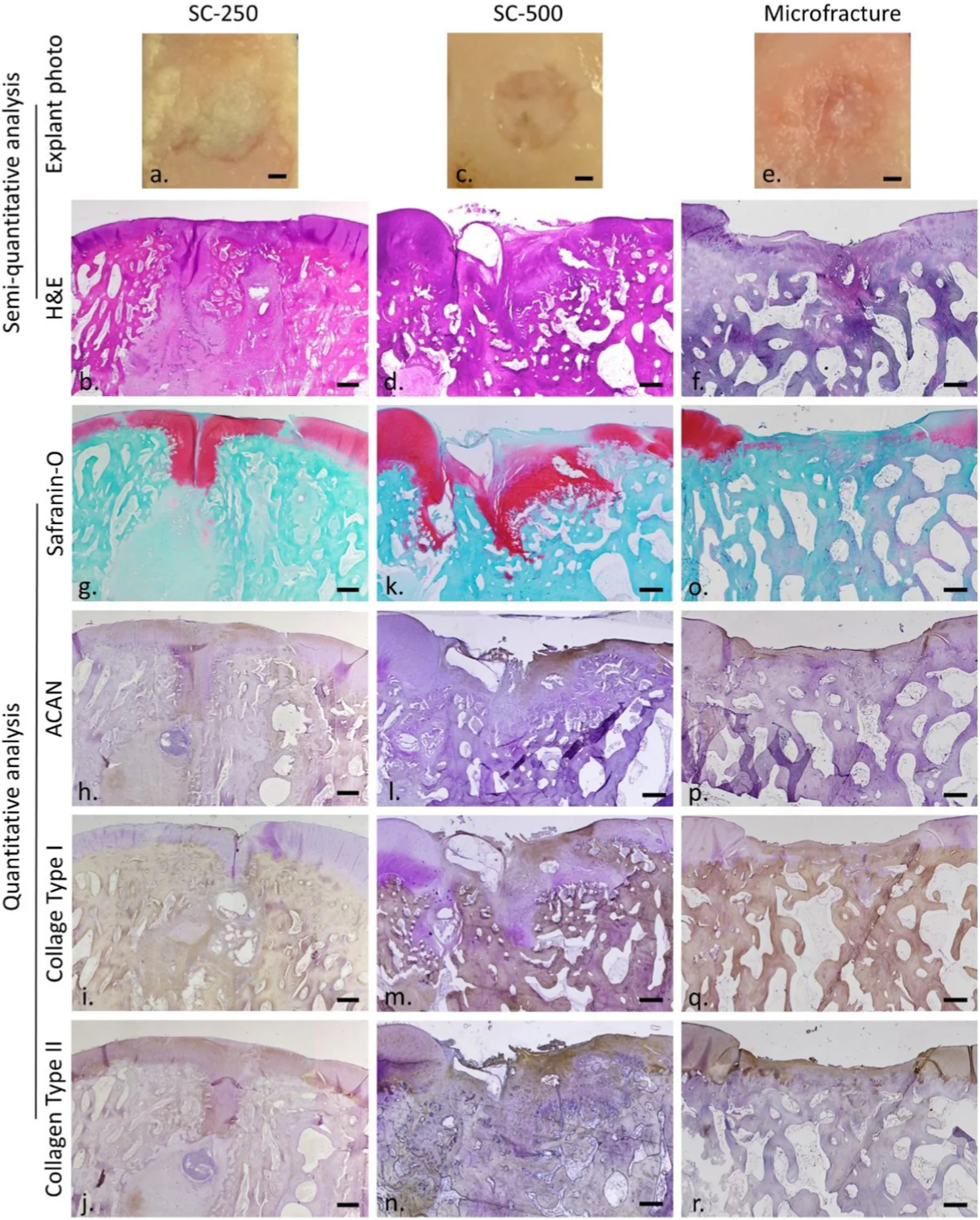
Explant photos, histology, and immunohistochemistry for semi-quantitative (a-f) and quantitative evaluation (g-r) of the chondral defects after 3 months *in vivo* in the ovine model, for each condition: scaffold with 250 μm pores (SC-250); scaffold with 500 μm pores (SC-500); and microfracture. Quantitative analysis was performed with: Safranin-O stain and Fast Green counterstain to identify cartilage in red and bone in green; ACAN to identify aggrecan; Collagen type I and II to identify the type of collagen formed. Scale bar explant photos 1 mm and histology micrographs 500 μm.

SC-250 resulted in high quality tissue regeneration. The regenerated tissue filled the defect to the level of the original articular surface and integrated with the surrounding tissue, bonding laterally to the healthy cartilage and integrating vertically downwards into subchondral bone (Fig. 3 SC-250: a, b). The tissue regenerated also presented the correct internal structure: a superficial zone with elongated chondrocytes parallel to the articular surface [37], and a vertical alignment of the chondrocytes was present in the deep zone [37]. The cells had the correct morphology, a mix of oval and round shape. The tidemark delimitating the cartilage to the subchondral bone was partially formed, evident from the incomplete calcification and transitional zone [37] within the cartilage. Both these two partial formations are a sign of the in-progress maturation of articular cartilage. The additional cartilage ingrowth into the subchondral bone was due to the microfracture performed during the surgery.

The implantation of SC-500 resulted in less regeneration of tissue, causing a counter sunk top surface compared to the neighbouring cartilage and a visible interface formed between the defect and surrounding cartilage (Fig. 3 SC-500: c, d). With regards to the tissue quality, some oval and round chondrocytes were visible, however, so was fibrous tissue and areas of hypocellularity. The tissue structure had no obvious organisation visible, with a superficial zone only starting to form and fissures on the articular surface. The adjacent cartilage was affected by the implantation of SC-500 and had regions of hypocellularity, and large clusters of chondrocytes, a sign of tissue degeneration [38]. Overall, the regenerated tissue was well adhered to the subchondral region, however, improper calcification was observed at the interface and no signs of tidemark was visible.

Our microfracture control showed poor tissue regeneration, only a thin layer of fibrous tissue formed, and some subchondral bone was still exposed (Fig. 3 microfracture: e, f). Furthermore, the tissue formed showed no orientation: the chondrocytes were elongated, individual chondrocytes and areas of hypocellularity were present. The adjacent cartilage also showed hypocellularity and chondrocytes clustering, sign of tissue degeneration. One positive was that the tissue formed was well anchored at the lateral and basal ends. However, in the subchondral region, microfracture caused amorphous tissue formation and cysts occurred deeper in the bone in approximately 1 out of 3 animals.

According to this qualitative assessment, SC-250 triggered more tissue regeneration compared to SC-500 or microfracture. SC-250 also promoted an improved tissue structure and quality, similar to that expected for articular cartilage. Further histology and immunohistology were then performed to qualify the tissue regenerated by SC-250, SC-500, and microfracture alone.

### 2.4 Semi-quantitative cartilage regeneration in ovine model

Scoring methods are commonly used to assess the morphology and quality of the regenerated tissues. Outerbridge classification is a visual scoring of the articular cartilage lesion [39] performed in this study by the surgeon immediately prior to explantation. Existing semi-quantitative scoring methods, such as the modified O'Driscoll [40] and ICRS II [41], were devised for osteochondral defects and were not appropriate for this study. Here, only chondral defects are considered, which have an implanted biomaterial that gradually degraded and was progressively replaced by cartilage tissue. Consequently, direct validation of our findings using the ICRS II framework was not feasible. Therefore, we devised a new bespoke semi-quantitative scoring method based on these existing methods (called HTGJ, Table 1) to assess the quality of the regenerated cartilage from H&E analysis. Our new scoring method assesses the chondral tissue for regeneration, while the surrounding tissues (native cartilage and subchondral bone) were evaluated for negative impact from the procedure. It was performed on H&E-stained explants sections, examples in Fig. 5f, with the detailed values breakdown in Fig. 4. The results were obtained using four independent assessors that evaluated the tissue within and surrounding the defect. While minor variation in scoring was observed, there was overall agreement among the assessors.

**Table 1:** **HTGJ scoring method for semi-quantitative analysis of chondral defects based on the modified O'Driscoll** [40] **and ICRS II** [41] **established scoring methods, total of 31 points modelled from native articular cartilage.**

Structural characteristics		
Characteristics	Outcomes	Score
Surface irregularities	smooth intact	3
	superficial horizontal lamination (<25 %)	2
	fissures 25-100 % thickness	1
	severe disruption with fibrillation	0
Thickness	75-100 %	3
	50-75 %	2
	25-50 %	1
	0-25 %	0
Width filling	100 % fully connected	3
	> 50 % partially growing	2
	≤ 50 % partially growing	1
	not growing	0
Bonding to adjacent cartilage	bonded at both ends	3
	bonded partially at both ends	2
	bonded partially at one end	1
	not bonded	0
Basal integration	full	2
	partial	1
	none	0
Formation of tidemark	single tidemark	2
	slight to moderate calcification/tidemark	1
	no visible interface or wrong place	0
Collagen architecture	3 zones of proportions appropriate for hyaline cartilage	3
	3 zones present	2
	deep zone well developed (vertical fibres)	1
	no organisation	0
Subchondral abnormalities	normal	3
	increased remodelling	2
	bone necrosis/cyst	1
	detached/fracture	0
Abnormal calcification/ossification/vessel in cartilage	none	2
	visible near interface with bone	1
	inside cartilage/inadequate cartilage regenerate to comment	0
**Freedom from cellular changes of degeneration**		
**Characteristics**	**Outcomes**	**Score**

Cell morphology	round/oval	2
	mix	1
	no round cells	0
Chondrocyte clustering	columnar	3
	mixed	2
	individual/fibro tissue	1
	hypocellular	0
**Freedom from degenerative changes in adjacent cartilage**		
**Characteristics**	**Outcomes**	**Score**

Degenerative changes in adjacent cartilage	normal	2
	mild clusters/hypocellularity/fissures	1
	hypocellularity	0
**Total**		**31**

**Fig. 4:**
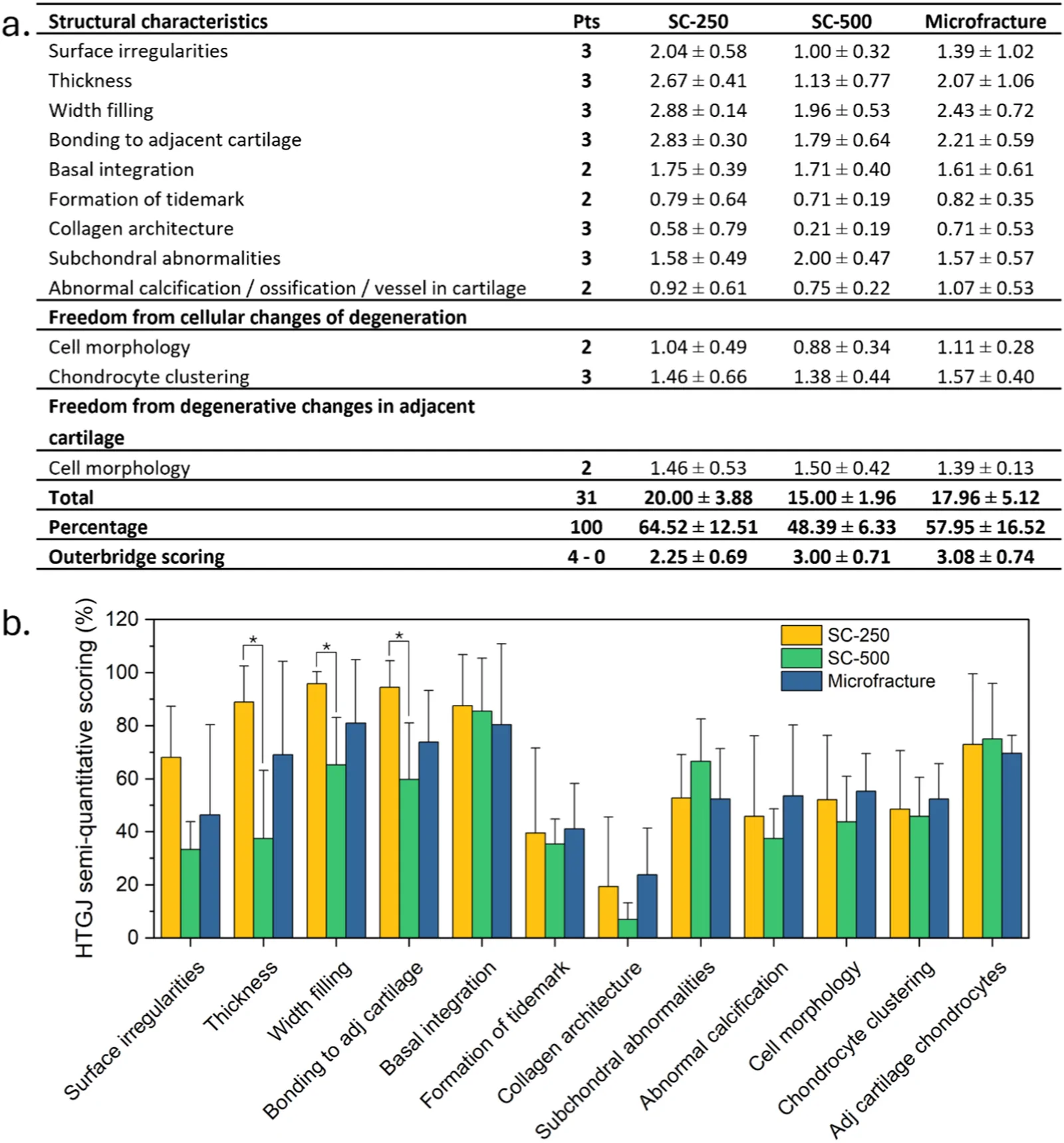
a. Semi-quantitative results of the HTGJ scoring system of defect sites for SC-250, SC-500, and microfracture (where a high score shows better performance) and Outerbridge grades (grade 0, normal; grade 1, surface fibrillation; grade 2, < 50 % loss cartilage thickness; grade 3, > 50 % loss cartilage thickness; grade 4, exposed subchondral bone) [39], b. visual representation of the HTGJ scores. Scores are reported as mean ± standard deviation (the error bars represent the standard deviations), the maximum number of points was 31 for the HTGJ scoring. A One-way ANOVA with Tukey Post Hoc multiple comparisons test was used to detect statistical significance of the structural characteristics between implants (SC-250, SC-500 and microfracture), ns p > 0.05, *p ≤ 0.05, **p ≤ 0.01, ***p ≤ 0.001. For clarity of the image only statistically different values are highlighted and “ns” is the value obtained for all the other pairs.

**Fig. 5:**
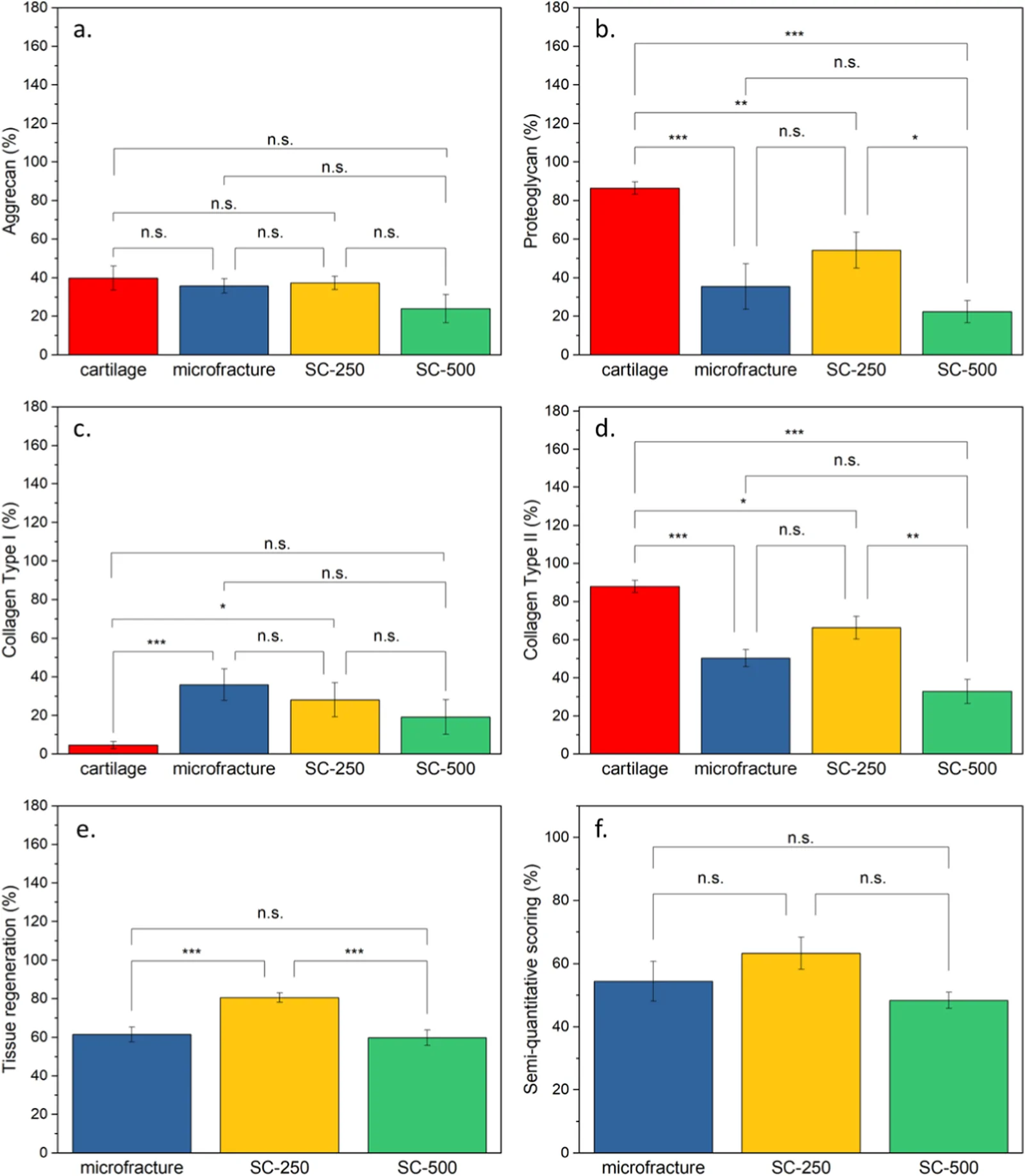
Quantitative analysis of the percentage of staining for the 250 μm channel scaffolds (SC-250), 500 μm channel scaffolds (SC-500), microfracture, and a positive control of native articular cartilage, representative of a. aggrecan (ACAN), b. proteoglycan (Safranin-O, Fast green), c. collagen type I, d. collagen type II, and e. the amount of tissue regenerated. f. semi-quantitative scoring as a percentage based on the HTGJ scoring system. A One-way ANOVA with Tukey Post Hoc multiple comparisons test was used to detect statistical significance between conditions, ns p > 0.05, *p ≤ 0.05, **p ≤ 0.01, ***p ≤ 0.001. Error bars are the standard error of the mean.

SC-250 scored the highest using the HTGJ scoring method, at 20/31, outperforming SC-500 and microfracture by approximately 15 % and 10 %, respectively (Fig. 4a). In agreement with the Outerbridge scores given upon explantation: SC-250 was close to grade 2 (<50 % loss cartilage thickness), whereas SC-500 and microfracture both graded 3 (>50 % loss cartilage thickness) (Fig. 4a). Based on the amount of tissue formed, SC-250 scored the highest in the surface irregularities, thickness, width filling, and bonding to adjacent cartilage criteria. The thickness, width filling and bonding to adjacent cartilages scores for SC-250 were significantly higher than the ones for SC-500. The microfracture and SC-500 groups both scored lower and had higher variability. In contrast, the tissue quality scored similarly for all three conditions based on the basal integration, formation of a tidemark, collagen architecture, subchondral abnormalities, abnormal ossification, cell morphology, chondrocyte clustering, and cell morphology criteria. Taken together, these results confirm the superior potential of SC-250 to regenerate tissue, although the 3-month timeframe may not be sufficient to reach cartilage maturation.

### 2.5 Quantitative cartilage regeneration in ovine model

The histology and immunohistology stains of the explants (Fig. 3 SC-250: g, h, i, j; SC-500: k, l, m, n; microfracture: o, p, q, r) were quantitatively analysed and summarised in Fig. 5. The percentage of positive stain for aggrecan, proteoglycan, collagen type I and II was calculated within the tissue regenerated (Fig. 5a, b, c, and d) and compared for 4 conditions: SC-250, SC-500, microfracture, and positive control of native articular cartilage as the healthy baseline. Native articular cartilage has high collagen type II content, which provides shock absorbing properties, whereas fibrocartilage is rich in collagen type I [37].

In our immunohistochemistry analysis, the collagen type II, an abundant protein in articular cartilage [37], indeed measured the highest in our native cartilage control, at over 81 %. Closely following, the regenerated tissue from SC-250 contained 66 % of collagen type II, just 15 % less than native cartilage (p ≤ 0.05). In contrast, the tissue resulting from SC-500 implantation contained only 30 % collagen type II, significantly lower than the tissue regenerated by SC-250 (p < 0.01). Finally, microfracture contained 50 % collagen type II, significantly lower than native articular cartilage (p < 0.001) (Fig. 5d). Collagen type I, typical of fibrocartilage [37], was then investigated: the tissue regenerated by SC-250 and SC-500 contained approximately 30 % and 20 % collagen type I, respectively. Microfracture produced the highest percentage of collagen type I, reaching 40 %. In contrast, the collagen type I content of native articular cartilage was significantly less, at 5 % (Fig. 5c). Fibrocartilage is composed of 80 – 90 % collagen type I [42], therefore, none of the tissue regenerated can be concluded to be mature fibrocartilage. Cartilage regenerated within the defect treated with SC-250 contains a high proportion of collagen type II, although smaller amounts of collagen type I are also present. This represents a considerable improvement compared to microfracture treatment and it is considered evidence that the regenerated tissue by SC-250 is maturing into articular cartilage (in line with *in vitro* studies). Similarly, cartilage repair following autologous chondrocyte implantation (ACI) initially includes collagen type I; however, this tissue has been shown to undergo gradual maturation, with increasing production of collagen type II over time [43]. There is no clear reason why a comparable remodelling process, as observed in other applications, should not also occur when SC-250 is used.

Aggrecan is a major proteoglycan, a glycosylated protein, in articular cartilage. It provides load-bearing properties through a hydrated gel structure from its interaction with hyaluronan and link proteins [44]. The difference in the amount of aggrecan was non-significant for all four conditions. However, a 20 % net difference in the average values of aggrecan between SC-250 and SC-500 was measured. Additionally, SC-250 matched the positive control and microfracture (Fig. 5a). Proteoglycans provide cartilage with its osmotic properties and its ability to resist compressive loads. They interact with collagen, are much smaller than aggrecan in size and are present in similar molar amounts [45]. Fibrocartilage contains low concentration of proteoglycans, limiting its shock absorption capabilities [46]. In contrast, proteoglycans are up to 30 % of the articular cartilage dry weight [45]. While the native cartilage had a much higher content of proteoglycan than any of the regenerated tissue, the difference was smaller for SC-250 (p < 0.01) than for the SC-500 and microfracture (p < 0.001) (Fig. 5b). The SC-250 promoted a net increase in the average value of proteoglycan content of 30 % when compared to SC-500 and of 20 % when compared to microfracture (Fig. 5b).

One of our most significant metrics investigated was the overall amount of tissue regenerated, which was calculated as a percentage of the total defect area (Fig. 5e). SC-250 promoted 80 % regeneration of the defect site, which was significantly more than for SC-500 (60 %) and microfracture (62 %) (p < 0.001). Altogether, the quantitative analysis of the histology and immunohistochemistry results demonstrated that despite a limited timeframe of 3 months, SC-250 promoted higher quantity and quality of regenerated tissue, characterised by a higher amount of collagen type II which is a key identifier for articular cartilage.

These quantitative analysis results also validated the new HTGJ scoring method. For instance, the individual implant that achieved the highest scores was a SC-250 implant (Fig. 6). Visually, healing of the defect was good (Fig. 6a–c), scoring 2 in the Outerbridge classification, within the average score for SC-250 (2.25 ± 0.69, Fig. 4a). There was no bone cyst or increased remodelling visible (Fig. 6d). This sample was the only one in which the articular cartilage zonal structure fully formed, clearly showing the superficial, transitional, and deep zones (Fig. 6e). Collagen type I was the lowest of all the implants (Fig. 6i) and informs the lack of fibrocartilage formed. In evidence of articular cartilage forming, proteoglycan content (toluidine blue and safranin-O/fast green, Fig. 6f–g) was similar in the regenerative cartilage compared to the control native. The proteoglycan, aggrecan, and collagen type II contents (Fig. 6g, h, j) were within the average of other SC-250 implants and higher than SC-500 or microfracture.

**Fig. 6:**
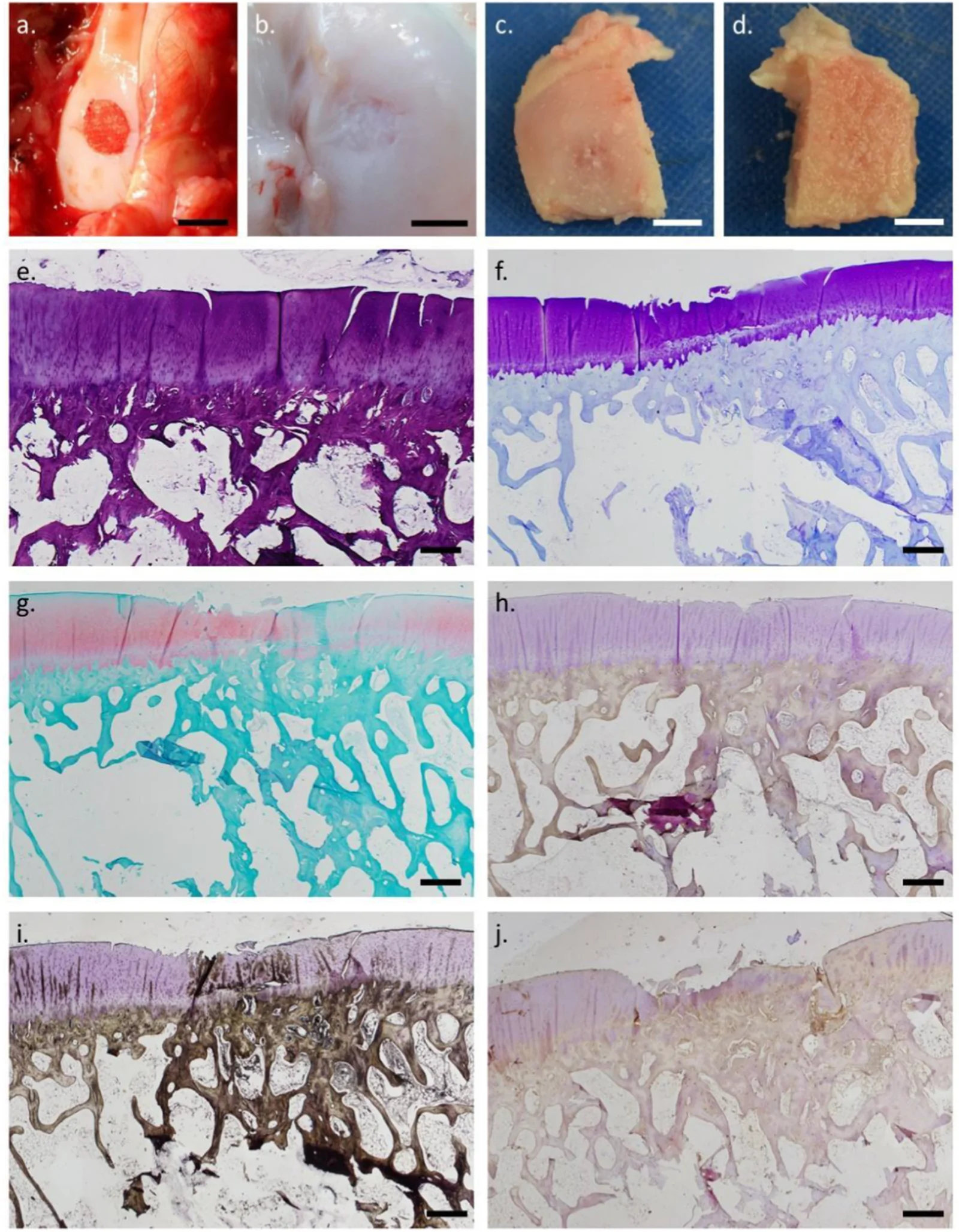
The SC-250 implant showing the best overall scores and results. Photos of this implant a. immediately after implantation, showing good marrow uptake into the pores, b. *in situ* before explantation, c. explant with the regenerated tissue, d. underside of the explant showing bone with no sign of cyst. e. H&E staining showed normal and homogeneous cartilaginous tissue throughout with appropriate thickness and filling. Homogeneous subchondral tissue was observed. The visible vertical fissures from the superficial to the middle zone are artefacts due to the dehydration and slicing process. Histology and immunohistochemistry stains were used to measure the presence of specific proteins: f. Toluidine blue (proteoglycans), g. Safranin-O and Fast green (proteoglycans in red), h. ACAN (aggrecans), i. Collagen type I, and j. Collagen type II. Scale bar implant and explant photo 5 mm and histology micrographs 500 μm.

## 3 Conclusions

Our Bouncy Bioglass SC-250 performed better than the clinical standard of microfracture, promoting superior cartilage regeneration. Bouncy Bioglass showed no systemic cytotoxicity in the rat model with all visceral organs presenting a normal morphology. Our SC-250 implant in an ovine model promoted a significantly higher volume of tissue regeneration, almost filling the defect site, compared to SC-500 or microfracture. SC-250 also regenerated an organised articular cartilage zonal structure with round chondrocytes. The main evidence of articular cartilage formation was the averagely higher collagen type II than either SC-500 or microfracture. Our new HTGJ scoring system developed specifically to analyse chondral defects was validated by the quantitative analysis and demonstrated the potential for cartilage regeneration of the SC-250 implant. These promising results show the potential of Bouncy Bioglass SC-250 to treat chondral defects and regenerate good quality articular cartilage.

## 4 Materials and methods

### 4.1 Materials

All materials were obtained from Merck UK and VWR UK, unless specified otherwise.

### 4.2 Hybrid synthesis

Silica/poly(tetrahydrofuran)/poly(ε-caprolactone) (SiO_2_/PTHF/PCL-diCOOH) sol-gel hybrids were synthesised following the protocol detailed by Tallia et al. [26]. The first step was to synthesise dicarboxylic acid (PCL-diCOOH) from PCL diol (M_n_ = 530 Da) *via* the 2,2,6,6-Tetramethyl-1-piperidinyloxy (TEMPO) oxidation process [26,47]. This PCL-diCOOH (1 mol) was then dissolved in tetrahydrofuran (THF, 100 mL mg^−1^ with regards to PCL-diCOOH), followed by the addition of (3-glycidyloxypropyl) trimethoxysilane (GPTMS, 2 mol), and boron trifluoride diethyl etherate (BF_3_ᐧOEt_2_, 0.5 mol). This organic precursor solution was mixed for 1.5 h at room temperature, allowing the *in-situ* polymerisation of THF to occur, forming PTHF. In parallel, the inorganic solution was prepared by hydrolysing tetraethyl orthosilicate (TEOS:PCL-diCOOH, 80:20 wt%) using stoichiometric volume of deionised water (i.e., 4 mol of water per mole of TEOS and 3 mol of water per mole of GPTMS) in acidic condition with hydrochloric acid (1:3 % v/v deionised water, 1 M HCl). Once hydrolysed, the inorganic silica precursor solution was added dropwise to the organic solution and stirred at room temperature for 30 min, in a sealed perfluoroalkoxy alkane (PFA) container, to form the hybrid sol. Further THF evaporation was achieved by opening the lid under continuous stirring for a further 30 min to accelerate the gelation process and achieve appropriate viscosity for 3D printing. This hybrid ink was then transferred into 3 mL Luer-lock syringes and stored at −82 °C.

### 4.3 Hybrid 3D printed scaffolds

This hybrid ink was 3D printed using Direct Ink Writing at room temperature (18-22 °C) and humidity between 30 and 50 %. The ink was thawed at room temperature (10 – 15 min) and loaded into a Direct Ink Writing machine (Robocaster, 3d Inks LLC, USA) with a tapered tip (Nordson EFD, UK). The software RoboCAD (3d Inks LLC, USA) was used to control the printing parameters to create a porous scaffold. Due to the continuous gelation process of the sol-gel hybrid ink, the correct printing viscosity had a printing window of 1 h where it allowed for smooth extrusion and no collapsing of struts once printed. For each 3-mL syringe, printing was commenced when viscosity was sufficient to retain filament dimensions during extrusion and the 1 h printing window allowed to print the full volume of the syringe, which normally led to minimum four scaffolds of the targeted shape in this work. A tapered nozzle with internal diameter of 0.20 mm, a strut spacing of 0.60 mm or 1 mm for final channel size of 250 or 500 μm, SC-250 and SC-500, respectively, z-spacing of 0.21 mm, and printing speed of 10 mm s^−1^ were used. The scaffolds were printed with side dimensions of 10.8 mm and 12 mm for the SC-250 and SC-500, respectively, and varying number of layers 8 – 16 to achieve a final thickness between 0.8 and 1.2 mm for the ovine surgery, while taller implants with a final thickness between 2 and 2.5 mm were produced for the *in vitro* biodegradation study and the *in vivo* rat study For the mechanical characterisation involved in the biodegradation study, SC-250 scaffolds of 36 layers were 3D printed. These were then aged and dried in a 40 °C oven for 3 and 7 days, respectively. Once dried the samples were cut to 5 mm diameter scaffold discs using biopsy punches (Kai Medical, Japan) for the *in vivo* studies, while 5 × 5 mm squares were manually cut with a razor blade for the *in vitro* biodegradation study. Scaffolds were then washed in deionised water three times for 10 s to remove by-products. Scaffolds for *in vivo* studies were transferred to glass vials (Supelco, Inc, UK) and sterilised with 50 kGY γ-irradiation, according to a previously established protocol [30].

### 4.4 *In vitro* biodegradation study

To assess the *in vitro* degradation (up to 6 months) and its effect on mechanical properties, scaffolds were soaked in PBS and placed in an orbital shaker at 37 °C, 120 rpm to simulate the biodegradation environment in the human body. PBS solution was prepared by adding 5 tablets (P4417-50TAB, Sigma-Aldrich) in 1 L of deionised water. The pH of obtained solution was checked to be around 7.4. The volume of PBS solution was calculated at a ratio of 1 mL per 1.5 mg of the sample.

Inductively coupled plasma optical emission spectroscopy (ICP-OES, iCAP 6000 Duo equipped with iTEVA software) was used to measure the Si concentration in solution from the scaffold degradation. With previous experience of Si dissolution study investigated on SC-250 hybrid scaffolds [26], the concentrations of silica released detected were never over 10 μg mL^−1^. Therefore, the silica standards were made with concentrations of 0, 0.1, 0.2, 0.4, 0.8, 1, 5, 10, 20, 40 μg mL^−1^, predicted to sufficiently cover the range of silica released in 6 months. The calibrations were successful with high correlations values. For the present study, four replicates of each type (SC-250 and SC-500) were analysed at each time point (1 day, 3 days, 7 days, 1 month, 3 months, 6 months). PBS solutions were refreshed after each time point so that the measured concentration of Si released was the exact amount between two time points.

Fourier-transform infrared spectroscopy (FTIR, Thermo Scientific Nicolet iS10 FTIR equipped with Smart Golden Gate and OMNIC software) was used in attenuated total reflectance (ATR) mode for characterising the contents in samples without soaking, soaking for 1 month and 6 months. Sample preparation was done by manually grinding scaffolds into a powder using pestle and mortar. FTIR was set to run 64 scans with a resolution of 4 LP/mm. Absorbance spectra ranging from 4000 to 400 cm^−1^ were collected.

Liquid-state Proton Nuclear Magnetic Resonance (^1^H NMR) was used to determine the organic components in the degradation products. A JEOL 400 MHz spectrometer was employed for this study. PBS solutions taken from SC-250 and SC-500 samples soaked for 1 month and from the third to the sixth month were freeze-dried then diluted with a small amount (not quantitatively) of deuterated chloroform (CDCl_3_) so that the final solutions were more concentrated than the original ones. After the mixture was stratified, solid residues not soluble in chloroform precipitated at the bottom, while the supernatant was transferred into NMR tubes. PBS solution that had never been used for soaking was also characterised to be used as a baseline and to exclude peaks that are not due to the degradation of the samples.

The inorganic/organic (I/O) ratio of hybrid scaffolds was determined by Thermogravimetric Analysis (TGA, Netzsch Jupiter STA 449C combined with Proteus® software), where the burn-out mass corresponds to the organic component of the hybrid. SC-250 and SC-500 samples (∼12-15 mg) were prepared by manually grinding scaffolds with pestle and mortar at each time point. TGA was run on samples pre-soaking, and after immersion for 1 month and 6 months to assess the effect of soaking on the I/O ratio.

Scanning electron microscopy (SEM, JEOL JEM-6010plus/LA) was utilised in secondary electron scattering mode (SEI) with parameters of 15 kV as the accelerating voltage, a spot size of 60 and working distance (WD) around 11-18 mm. Samples (SC-250 and SC-500) for non-soaking, 1-month and 6-month soaking were coated with approximately 10 nm thick gold coating before imaging.

Compression test to fracture and dynamic mechanical analysis (DMA) were carried out with a Bose Electroforce Series III universal testing machine to evaluate the effect of the soaking in PBS on the mechanical performance of SC-250 scaffolds at 0, 1 month and 6 months (without refreshing of the PBS) in the shape of cubes with side dimension of 5 mm. Wet samples taken directly from PBS solutions were put in the centre of the plate placed on top of the loadcell, and PBS solution was added during the test to prevent sample drying out and changing the mechanical properties. PBS solutions were added on non-soaked samples to eliminate the effect of the wet environment on the mechanical performance that was aside from the effect of soaking itself.

Compression tests were conducted on 5 replicates at each time point at the displacement rate of 0.5 mm min^−1^ using the 440 N load cell. As dimensions were measured for each sample, the conventional strain (*ε*_c_) and stress (*σ*_c_) were calculated at the point of failure by Eqs. (1) and (2):

(1) *ε*_c_ = Δ*l*/*l*_0_

(2) *σ*_c_ = *F*/*A*

where *l*_0_ and *A* are, respectively, the height and top surface area defined by the sample, and Δ*l* and *F* are, respectively, the displacement and force applied when the material fails. Since ‘barrelling’ of the samples can be observed in compression, causing the area of the cross-section to vary, true strain (ε*) and stress (σ*) were calculated by Eqs. (3) and (4):

(3) ε* = ln(1+ *ε*_c_)

(4) σ* = *σ*_c_ (1+ *ε*_c_)

Empirically, Bouncy Bioglass' response in compression is elastomeric, meaning it can be completely recovered but the Young's modulus increases as the strain increases, so it cannot be calculated from the stress-strain curve. Hence, the storage modulus (E′), which is another measure of the stiffness of the samples, was determined by stress-strain gradient locally in small ranges through DMA. DMA was conducted in four ranges of strain (1-5 %, 5-9 %, 9-13 %, 13-17 %) where the stress-strain curve was nearly linear, 5 replicates for each condition were tested. The amplitudes of sine waves were constrained in ± 2 % of the equilibrium points of strain (3 %, 7 %, 11 %, 15 % respectively). A load cell of 220 N was used. For each sample, 3 frequencies were tested (0.1, 1, 10 Hz). The storage modulus is related to the energy stored in the elastic portion of the material while the loss modulus (E″) is contributed by the viscous portion that the energy dissipates *via* heat. The loss tangent (tan(*δ*)) is the ratio of the two expressed as below (Eq. (5)):

(5) tan(*δ*) = *E*’’/*E*’

representing how large the extent of loss modulus playing a role in the material [48].

### 4.5 *In vivo* rat study

The objective of this study was to investigate the cytotoxicity of Bouncy Bioglass using the SC-250 scaffold (n = 6) and to compare the results with a standard UHMWPE control (n = 3), using a total of n = 9 Wistar male rats. The implants (5 mm diameter, 2 mm thick) were buried in the sacrospinous muscle adjacent to the lumbar vertebrae for a 6-week duration. The general health of the animals, including grimace scoring and weight change, was observed daily by an experienced animal technician at the RVC.

Anaesthesia was carried out *via* inhalation. Rats were induced by gaseous isoflurane in an anaesthetic chamber, surgical site clipped and sterilised according to establishment's protocol and anaesthesia maintained on gaseous isoflurane *via* nose cone. The dorsal aspect overlying the lumbar spine was shaved, cleaned and prepared for surgery. The animal was moved into the surgical area and positioned on a heating mat to keep the animal warm. Using aseptic techniques, the shaved area was cleaned again using a proprietary surgical preparation. Through a small incision 1 cm on the left side of the spine the sacrospinalis muscle was exposed. Using blunt dissection, a small pocket was created in the proximal direction, large enough to enclose the 5 mm diameter and 2 mm thick implants. The fascia was closed using a 3-0 resorbable suture (PDS) and the skin was closed using a Monocryl 3-0 subcutaneous suture. Bupivacaine was infiltrated into the wound and around the skin margins prior to closure. A combination of meloxicam at 1 mg kg^−1^ and buprenorphine at 0.05 mg kg^−1^ was given at the time of surgery *via* the subcutaneous route and in accordance with each animal's weight. Analgesia was administered pre and postoperatively by administration through subcutaneous injections over a period of 3 days postoperatively. Two subcutaneous injections of meloxicam at 1 mg kg^−1^ were provided at 24-h intervals as post-operative analgesia (days S1 and S2). If signs of pain were noted at 72-h time point (day S3), subcutaneous meloxicam pain relief continued. If at any point sufficient pain relief on meloxicam alone was not observed, then addition of buprenorphine was considered and confirmed with veterinary surgeons.

To extract the relevant results, tissue formation around both materials implanted was evaluated using Masson Trichrome and their cytotoxicity was investigated by examining the H&E stains of the retrieved organs (kidney, liver, lung, spleen, and heart). The organs were weighed immediately after removal. Using the open-source software QuPath (v 0.4.2) for bioimage analysis [49] and the FIJI software, the stains were analysed to evaluate the specific structure of each organ, ensuring no morphological changes or pathological lesions.

### 4.6 *In vivo* ovine study

The objective of this study was to test the regenerative capacity of the 3D printed SiO_2_/PTHF/PCL-diCOOH hybrid in an ovine model (n = 9 sheep with 2 condyle implant sites). This study compared two types of implants: the SC-250 (n = 6) and SC-500 (n = 6), Fig. 7. The control used was microfracture in an empty defect (n = 6), the current articular cartilage repair gold standard. The ideal implantation method is simply by press-fitting the implant into a defect resected to match its size. However, sheep cartilage being so thin in the stifle condyle, between 0.8 and 1.2 mm [50,51], additional fixation was used with a periosteal flap (PF) kept in place with cyanoacrylate glue.

**Fig. 7:**
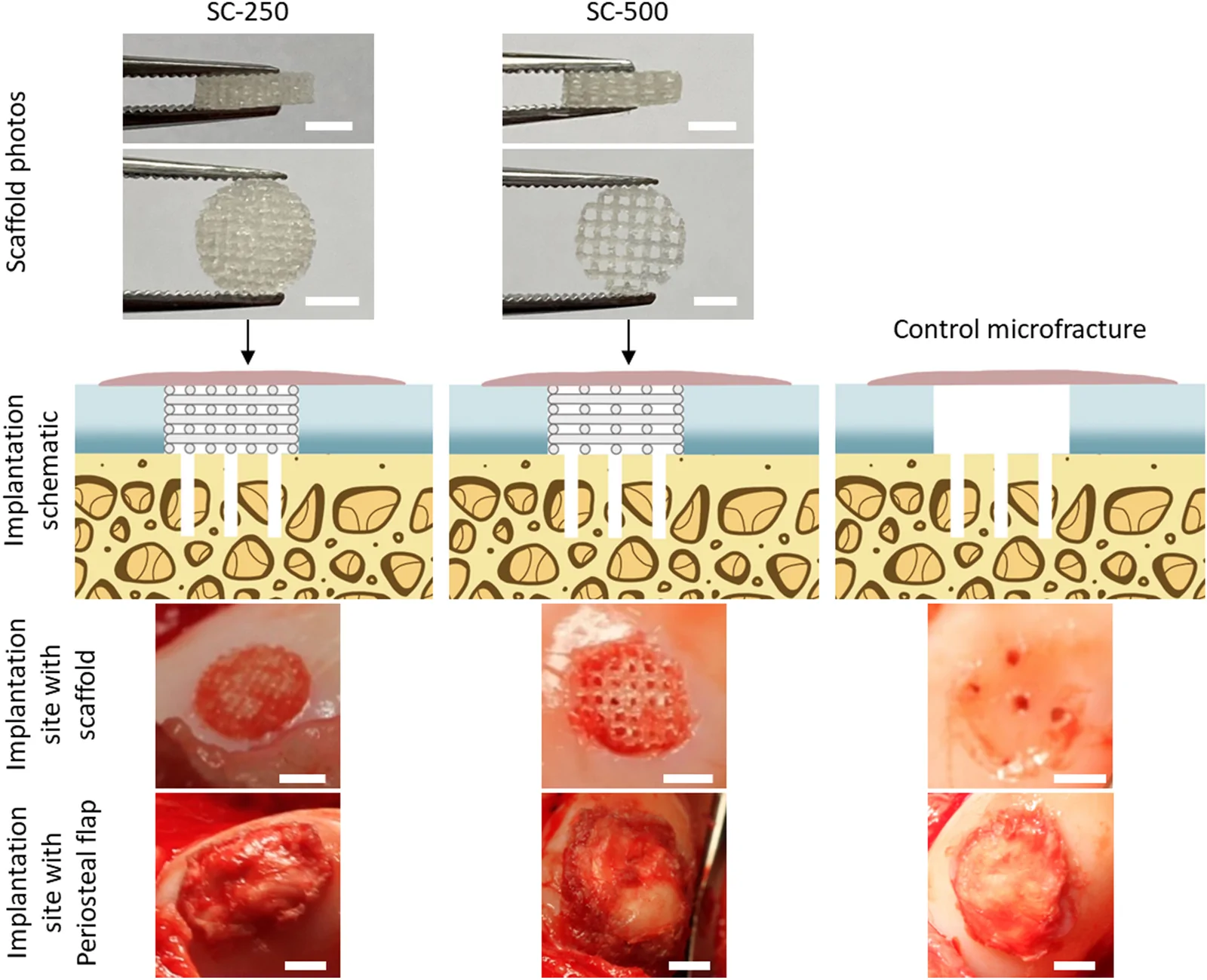
Schematic of the ovine study implants and fixation methods investigated, 250 μm chondral scaffolds (SC-250, left column), 500 μm chondral scaffolds (SC-500, middle column), and empty control defect (right column). All have been microfractured and fixed with cyanoacrylate glue and a periosteal flap (PF). Photo scale bars 2 mm.

### 4.7 Preoperative

12 h prior to the surgery and 60 h after, Durogesic™ topical analgesic patches (75 mg Fentanyl each), were administrated. Anaesthesia was induced with a combination of ketamine (100 mg mL^−1^) and midazolam (5 mg mL^−1^), intravenously. Anaesthesia was maintained using intubation with sevoflurane and oxygen. A gastric tube was used to evacuate gas and fluid content in the rumen. After clipping and preparing the surgical site, the animal was moved to the operating theatre. Prior to surgery, and for three days postoperatively, antibiotic injections (penicillin/streptomycin) were given.

### 4.8 Operative technique

All operations were performed by the same lead surgeon [TAJ] using aseptic techniques (Fig. S8). A skin incision of 5 – 10 cm long and longitudinal located in the medial side of the patella was made, and the patella was dislocated laterally. Both the medial and lateral condyle (MC and LC) were exposed and visible by flexing the stifle to 70 - 90°. The defects were made in the thickest part of the cartilage on both the medial and lateral condyles of the left stifle of the animal, in a load-bearing region. Using a 5 mm diameter disposable biopsy punch (Kai Medical, Japan), a defect was created in the medial and lateral condyle cartilage, the circular defect was then debrided using a dermal curette and a micro curette (Arthrex, Germany). The thickness of the defect was measured, to choose the most suited implant thickness. The implants were placed in saline solution, as filling the pores with saline pre-implantation enabled the pores to fill with the microfracture blood and fat. To create the microfracture, the subchondral bone plate was pierced through using a 1.0 mm K-wire and electric drill (Bosch, Germany), no deeper than 5 mm. Then a 0.8 mm green needles and a clip were used to manually create the 10 mm deep microfracture. The holes were made at positions 12, 3, 6, 9 o'clock and at the centre of the defect. Blood and fat from the bone marrow filled the defect from the microfracture. Before implanting the scaffolds, saline and suction were used to clean the area of any fragments.

The implants were placed in the defects by press-fitting, ensuring they were not proud. They were glued in place with cyanoacrylate Derma + flex glue (Chemence Medical Inc., US), placing three small dots of glue at the edges of the implant, in contact with the healthy cartilage. The periosteal flap (PF) was harvested from the medial distal femur for both the MC and LC defects. The 2 PFs were cut in more circular shapes, keeping it attached to the harvest site until ready to place over the implants and defect. A small amount of Derma + flex glue was placed on the inside of the PF (cambium layer, tissue layer with partially undifferentiated cells) at the outer edges. They were then placed on the implant, cell side in contact with the material. More glue was applied at the outer edge on top of the PF and then pressed down, smoothing out the edges. The finished implant site was left to dry for 5 min before relocating the patella and cycling the stifle joint 10 times gently, not going to full flexion. The wound was closed using 3 different types of sutures: (a) PSD, polydioxanone, for soft tissue and deep layers, such as muscles, (b) Vicryl for intermediate soft tissue, such as fascia tissue, and (c) Monocryl for top layers, such as the skin. 10 mL of local anaesthetic (Marcain Polyamp Steripack 0.5 %) was applied around the wound. The wound was then cleaned using swabs with saline, followed by Opsite spray-on dressing and Dermabond glue applied to the wound to further seal the surgery site. A dressing (Primapore) was placed and then cotton wool used to cover the entire top hind leg, which was finally wrapped using Vetrap.

### 4.9 Postoperative

Animals recovered in single pens on soft bedding adjacent to each other and allowed to weight bear freely and given food immediately after the procedure. The gait of the animal was observed; animals were expected to be standing after a few hours and weight bear within two days of the surgery. When fully weight bearing, they were group housed on straw in a large pen in a barn and given food *ad arbitrium.*

The animals were euthanised at the end of the study (3 months), and the tissues of interest harvested for analysis (medial and lateral of the left stifle per animal, n = 9). Outerbridge scoring was performed as soon as the defect sites were exposed, with grade 0, normal; grade 1, surface fibrillation with softening and swelling of the cartilage; grade 2, < 50 % loss cartilage thickness with small fissuring; grade 3, > 50 % loss cartilage thickness with large fissuring and no bone exposure; grade 4, full thickness chondral defect with exposed subchondral bone [39]. The implants were isolated and removed *en bloc* leaving approximately 5 mm of surrounding cartilage and 5 to 10 mm of underlying subchondral bone, using an oscillating saw. All harvested samples were placed in falcon tubes with PBS solution and stabilised antibiotic antimycotic solution of 1 % concentration for transportation.

### 4.10 Explant analysis

The samples were fixed in 10 % Formalin for 1 to 3 days. Thereafter, decalcification was done in a 20 % w/v ethylenediaminetetraacetic acid (EDTA) solution (pH 7.4), with intermittent shaking, and changing the solution two to three times a week (for a total of 4 weeks). The samples were then washed twice for 1 h in deionised water and then dehydrated in 70 %, 95 % and 100 % ethanol for 4 h in each solution. The final 100 % ethanol step was repeated 3 times. They were then cleaned in 3 changes of xylene (VWR, Lutterworth, UK) overnight, before being embedded into paraffin wax overnight at 58 °C, twice. The samples were then sliced using a Leica RM2235 rotary microtome (Leica Biosystems, Nussloch, Germany), to 7 μm thickness. Sections were histologically stained with Haematoxylin and Eosin (H&E), Safranin O/Fast green and Toluidine blue[52]. In the immunohistochemistry protocol, tissue sections underwent antigen retrieval with pepsin solution (20 min, 37 °C), followed by blocking of non-specific binding using 2.5 % bovine serum albumin. Subsequently, the sections were incubated overnight at 4 °C with primary antibodies diluted in PBS/BSA (0.5 %). The primary antibodies used were as follows: Collagen I (1:500) (ab138492, Abcam), Collagen II (1:250) (ab34712, Abcam), and Aggrecan (1:300) (ab3778, Abcam). Following rinsing, secondary antibodies were applied: anti-Rabbit for collagen I and II (ab205718) and anti-Mouse for aggrecan (ab6789), both diluted at 1:2000. Subsequently, sections were treated with DAB substrate (ab64238, Abcam) and incubated for 3 min for collagen I, 5 min for collagen II, and 10 min for aggrecan (Abcam, Cambridge, UK). Finally, counterstaining was performed using haematoxylin for 1 min. Then, samples were mounted with DPX and imaged using an Olympus BX53M Brightfield Darkfield Upright microscope and Stream Essential software.

H&E histology staining was analysed using a bespoke scoring system (Heyraud Tallia Gupte Jones, HTGJ), generated in this work, modelled on the modified O'Driscoll [40] and ICRS II [41] established scoring methods. All pre-established scoring methods were for osteochondral defects, which were not appropriate here. The new HTGJ scoring system, Table 1, was established for semi-quantitative analysis of chondral defect, here used for an ovine model. A comprehensive masking of slides was performed (individual samples labelled without reference to treatment group). Four independent scorers evaluated and scored each sample. Validation of repeatability of the scorers was ensured by scoring multiple slides for the same sample. A One-way ANOVA with Tukey Post Hoc multiple comparisons test was used to detect statistical significance of the structural characteristics between implants (SC-250, SC-500 and microfracture).

The immunohistochemistry was measured using the open-source software QuPath (v 0.4.2) for bioimage analysis [49], giving quantitative data for proportion of tissue stained for proteoglycans (Safranin-O), aggrecan (ACAN), and collagen type I and II. To get accurate quantitative data, three regions of interests were identified: the entire defect area (including any regenerated tissue and void, Fig. S9a), the regenerated tissue area only (Fig. S9b), and the unaffected neighbouring cartilage Fig. S9c). The entire defect area was used to calculate the percentage of tissue regenerated, whereas the regenerated tissue area was used to evaluate the quality of the tissue formed by quantifying the positive stain percentage within the tissue. Control data for each antibody was quantified from the non-affected cartilage areas (Fig. S9c). One-way ANOVA with Tukey Post Hoc multiple comparisons test was used to detect statistical significance between the two implants and control using Origin (OriginLab Corporation). The analysis was performed on the percentage stain measured using QuPath for the entire defect area, including the regenerated tissue and the defect void (Fig. S9c).

## CRediT authorship contribution statement

**Agathe Heyraud:** Writing – original draft, Methodology, Investigation, Formal analysis, Data curation. **Francesca Tallia:** Methodology, Investigation, Formal analysis, Data curation, Writing – review & editing. **Talal Al-Jabri:** Methodology, Investigation, Conceptualization. **Arianna De Mori:** Investigation, Formal analysis, Data curation. **Qingyue E. Shi:** Data curation, Investigation. **Diogo M.M. dos Santos:** Methodology. **Jeffrey N. Clark:** Methodology, Investigation, Formal analysis. **Chinmay Gupte:** Writing – review & editing, Methodology, Investigation, Funding acquisition. **Jonathan R.T. Jeffers:** Supervision, Methodology. **Eduardo H.M. Nunes:** Writing – review & editing, Supervision. **Gordon Blunn:** Writing – review & editing, Supervision, Methodology, Investigation, Funding acquisition, Formal analysis, Conceptualization. **Justin Cobb:** Writing – review & editing, Supervision, Methodology, Conceptualization. **Julian R. Jones:** Writing – review & editing, Writing – original draft, Supervision, Project administration, Methodology, Investigation, Funding acquisition, Formal analysis, Conceptualization.

## Ethics approval and consent to participate

The animal study protocol was approved by the Animal Welfare and Ethical Review Body at the Royal Veterinary College, UK and the procedures on animals was carried out under a UK Home Project Office License (License number P16F4AA0A, 5 Aug 2019).

## Declaration of competing interest

The authors declare the following financial interests/personal relationships which may be considered as potential competing interests:

Julian Jones reports financial support was provided by Engineering and Physical Sciences Research Council. Francesca Tallia reports financial support was provided by Wellcome Trust. Julian Jones reports a relationship with Orthox Limited that includes: consulting or advisory and equity or stocks. Francesca Tallia reports a relationship with Orthox Limited that includes: consulting or advisory and equity or stocks. Agathe Heyraud reports a relationship with Orthox Limited that includes: consulting or advisory and equity or stocks. Julian Jones has patent #WO2017168168 licensed to Orthox Limited. Francesca Tallia has patent #WO2017168168 licensed to Orthox Limited. If there are other authors, they declare that they have no known competing financial interests or personal relationships that could have appeared to influence the work reported in this paper.

## References

[1] Sherwood J.K. (2002). A three-dimensional osteochondral composite scaffold for articular cartilage repair. Biomaterials.

[2] McCann L. (2008). Tribological testing of articular cartilage of the medial compartment of the knee using a friction simulator. Tribol. Int..

[3] Widuchowski W., Widuchowski J., Trzaska T. (2007). Articular cartilage defects: study of 25,124 knee arthroscopies. Knee.

[4] Hunziker E.B. (2002). Articular cartilage repair: basic science and clinical progress. A review of the current status and prospects. Osteoarthr. Cartil..

[5] Erggelet C., Vavken P. (2016). Microfracture for the treatment of cartilage defects in the knee joint – a golden standard?. J. Clin. Orthop. Trauma..

[6] Richter D.L., Schenck R.C., Wascher D.C., Treme G. (2016). Knee articular cartilage repair and restoration techniques: a review of the literature. Sports Health.

[7] Brittberg M.D.M. (1994). Treatment of deep cartilage defects in the knee with Autologous Chondrocyte Transplantation. N. Engl. J. Med..

[8] Horas U., Pelinkovic D., Herr G., Aigner T., Schnettler R. (2003). Autologous chondrocyte implantation and osteochondral cylinder transplantation in cartilage repair of the knee joint. A prospective, comparative trial. J. Bone Joint Surg. Series A.

[9] Robert H. (2011). Chondral repair of the knee joint using mosaicplasty. Orthop. Traumatol.: Surgery and Research.

[10] Hangody L., Füles P. (2003). Autologous osteochondral mosaicplasty for the treatment of full-thickness defects of weight-bearing joints: ten years of experimental and clinical experience. J. Bone. Joint. Surg. - Series A.

[11] Melton J.T.K., Wilson A.J., Chapman-Sheath P., Cossey A.J. (2010). TruFit CB® bone plug: chondral repair, scaffold design, surgical technique and early experiences. Expert Rev. Med. Dev..

[12] Dunkin B.S., Lattermann C. (2013). New and emerging techniques in cartilage repair: MACI. Operat. Tech. Sports Med..

[13] Jacobi M., Villa V., Magnussen R.A., Neyret P. (2011). MACI - a new era?. Sports Med. Arthrosc. Rehabil. Ther. Technol..

[14] Behrens P., Bitter T., Kurz B., Russlies M. (2006). Matrix-associated autologous chondrocyte transplantation/implantation (MACT/MACI)-5-year follow-up. Knee.

[15] Piontek T., Ciemniewska-Gorzela K., Szulc A., Naczk J., Słomczykowski M. (2012). All-arthroscopic AMIC procedure for repair of cartilage defects of the knee. Knee Surg. Sports Traumatol. Arthrosc..

[16] Gille J. (2013). Outcome of Autologous Matrix Induced Chondrogenesis (AMIC) in cartilage knee surgery: data of the AMIC Registry. Arch. Orthop. Trauma Surg..

[17] Geistlich (2018). AMIC chondro-gide in the knee. Geistlich Surgery.

[18] Lee Y.H.D., Suzer F., Thermann H. (2014). Autologous matrix-induced chondrogenesis in the knee: a review. Cartilage.

[19] Erggelet C. (2009). Formation of cartilage repair tissue in articular cartilage defects pretreated with microfracture and covered with cell-free polymer-based implants. J. Orthop. Res..

[20] Buda R. (2010). Osteochondral lesions of the knee: a new one-step repair technique with bone-marrow-derived cells. J. Bone Joint Surg..

[21] Gobbi A. (2017). One-step surgery with multipotent stem cells and Hyaluronan-based scaffold for the treatment of full-thickness chondral defects of the knee in patients older than 45 years. Knee Surg. Sports Traumatol. Arthrosc..

[22] Ambrosi R.D., Valli F., Luca P. De, Ursino N., Usuelli F.G. (2019). MaioRegen osteochondral substitute for the treatment of knee defects : a systematic review of the literature. J. Clin. Med..

[23] Kon E. (2015). Osteochondral regeneration with a novel aragonite-hyaluronate biphasic scaffold: up to 12-month follow-up study in a goat model. J. Orthop. Surg. Res..

[24] Di Martino A. (2025). Osteochondral regeneration with a tri-layered biomimetic resorbable scaffold: in vivo study in a sheep model up to 12 months of follow-up. Biomaterials.

[25] Kon E., Conte P., Anzillotti G., Di Matteo B., Verdonk P. (2025). Report on evolving indications, techniques, and outcome of novel and innovative surgical procedure – agili C®. Curr. Rev. Musculoskelet. Med..

[26] Tallia F. (2018). Bouncing and 3D printable hybrids with self-healing properties. Mater. Horiz..

[27] Hodge W.A. (1989). Contact pressures from an instrumented hip endoprosthesis. J. Bone. Joint. Surg. - Series A.

[28] Heyraud A. (2023). 3D printed hybrid scaffolds for bone regeneration using calcium methoxyethoxide as a calcium source. Front. Bioeng. Biotechnol..

[29] Li S., Tallia F., Mohammed A.A., Stevens M.M., Jones J.R. (2020). Scaffold channel size influences stem cell differentiation pathway in 3-D printed silica hybrid scaffolds for cartilage regeneration. Biomater. Sci..

[30] Ferreira S.A. (2024). 3D printed hybrid scaffolds do not induce adverse inflammation in mice and direct human BM-MSC chondrogenesis in vitro. Biomaterials and Biosystems.

[31] De Mori A. (2024). Ovine mesenchymal stem cell chondrogenesis on a novel 3D-Printed hybrid scaffold in vitro. Bioengineering.

[32] Kaid F. (2019). Histological, biochemical, and hematological effects of goniothalamin on selective internal organs of Male sprague-dawley rats. J. Toxicol..

[33] Elblehi S.S., El-Sayed Y.S., Soliman M.M., Shukry M. (2021). Date palm pollen extract avert doxorubicin-induced cardiomyopathy fibrosis and associated oxidative/nitrosative stress, inflammatory cascade, and apoptosis-targeting bax/bcl-2 and caspase-3 signaling pathways. Animals.

[34] Niyomchan A. (2023). Safety evaluation of the polyherbal formulation NawaTab: acute and subacute oral toxicity studies in rats. Evid. Based Complement. Alternat. Med..

[35] Abd F. (2018). Amiodarone-Induced lung toxicity and the protective role of vitamin E in adult Male Albino rat. Eur. J. Anat..

[36] Rocha-Pereira C. (2019). Histological and toxicological evaluation, in rat, of a P-glycoprotein inducer and activator: 1-(propan-2-ylamino)-4-propoxy-9H-thioxanthen-9-one (TX5). EXCLI J..

[37] Fox A.J.S., Wanivenhaus F., Rodeo S.A. (2012). The basic science of Articular Cartilage: structure, composition, and function. J. Knee Surg..

[38] Lotz M.K. (2010). Cartilage cell clusters. Arthritis Rheum..

[39] Outerbridge R.E. (1961). The etiology of chondromalacia patellae. J. Bone Joint Surg..

[40] Getgood A. (2012). The augmentation of a Collagen/Glycosaminoglycan biphasic osteochondral scaffold with platelet-rich plasma and concentrated bone marrow aspirate for osteochondral defect repair in sheep: a pilot Study. Cartilage.

[41] Mainil-Varlet P. (2010). A new histology scoring system for the assessment of the quality of human cartilage repair: ICRS II. Am. J. Sports Med..

[42] Benjamin M., Evans E.J. (1990). Fibrocartilage. J. Anat.

[43] Roberts S., Menage J., Sandell L.J., Evans E.H., Richardson J.B. (2009). Immunohistochemical study of collagen types I and II and procollagen IIA in human cartilage repair tissue following autologous chondrocyte implantation. Knee.

[44] Kiani C., Chen L., Wu Y.J., Yee A.J., Yang B.B. (2002). Structure and function of Aggrecan. Cell Res..

[45] Roughley P.J., Lee E.R. (1994). Cartilage proteoglycans: structure and potential functions. Microsc. Res. Tech..

[46] Katta J., Pawaskar S.S., Jin Z.M., Ingham E., Fisher J. (2007). Effect of load variation on the friction properties of articular cartilage. Proc. IME J. J. Eng. Tribol..

[47] Anelli P.L., Biffi C., Montanari F., Quici S. (1987). Fast and selective oxidation of primary alcohols to aldehydes or to carboxylic acids and of secondary alcohols to ketones mediated by oxoammonium salts under two-phase conditions. J. Org. Chem..

[48] Ogoshi T., Chujo Y. (2005). Organic–inorganic polymer hybrids prepared by the sol-gel method. Compos. Interfaces.

[49] Bankhead P. (2017). QuPath: open source software for digital pathology image analysis. Sci. Rep..

[50] Frisbie D., Cross M., McIlwraith C. (2006). The quest for a better model: a comparative study of articular cartilage thickness on the femoral trochlea and femoral condyle of species used in pre-clinical studies compared to cartilage thickness in the human knee. Vet. Comp. Orthop. Traumatol..

[51] Ahern B.J., Parvizi J., Boston R., Schaer T.P. (2009). Preclinical animal models in single site cartilage defect testing: a systematic review. Osteoarthr. Cartil..

[52] Schmitz N., Laverty S., Kraus V.B., Aigner T. (2010). Basic methods in histopathology of joint tissues. Osteoarthr. Cartil..

