# Supplementary material for: 3D printed Bouncy Bioglass enhances articular cartilage regeneration
Source: Bioact Mater. 2026 Sep 22;69:162–76. doi: 10.1016/j.bioactmat.2026.09.023 (PMC13677033; doi:10.1016/j.bioactmat.2026.09.023)
Supplement: Supplementary file 1 [file mmc1.docx]

**Supplementary Information**

**3D printed Bouncy Bioglass enhances articular cartilage regeneration**

**Supplementary Figures for “Results and Discussion” Section**

**Dissolution of the inorganic component of the hybrid scaffolds using ICP-OES**

The dissolution profiles of Si show a general trend of sustained Si release for both types of hybrid scaffolds (Fig.S1). Fig. S1 presents the cumulative release of Si in PBS solution over time measured by ICP-OES. SC-250 and SC-500 scaffolds all continuously released Si over the 6 months (165 days) but with different rates. Scaffolds with 500 μm channels released Si fastest up to 13.22 ± 1.94 μg mL^-1^ after 6 months, whereas scaffolds with 250 μm channels reached 9.76 ± 1.54 μg mL^-1^. This is consistent with the larger pores corresponding to higher surface/volume exposed to the solution in the SC-500 scaffolds compared to the SC-250. The concentration of Si released in solution is still much lower than in sol-gel bioactive glass samples, where it can reach ~50 μg mL^-1^ after 2 weeks of soaking [1].

**Figure S1.** Cumulative release of Si from SC-250 and SC-500 scaffolds with refreshed PBS solution at each time point (n = 4). Error bars represent standard deviation.

**Evaluation of the hybrid network changes upon soaking**

FTIR spectra of SC-250 and SC-500 before and after soaking were comparable, confirming that the changes in architecture do not affect the hybrid network resulting from the synthesis. Fig.S2 shows example spectra from SC-250 scaffolds. Effects of soaking are expected to cause changes in the relative intensities of bands. The highest band in each sample is always the vibrational band of Si-O-C and Si-O-Si at around 1070 cm^-1^, representing the silica network (Fig.S2b). The main features of PCL are the narrow band at around 1725 cm^-1^ from stretching of C=O bond in carbonyl groups (Fig.S2d), while the double bands of symmetric and asymmetric stretching of CH_2_ at 2800-3000 cm^-1^ (Fig.S2c) and vibrations of CH_2_ at 1300-1500 cm^-1^ (Fig.S2d) are present in both PCL and PTHF. A clear reduction in the intensity of the PCL band at 1750 cm^-1^, relative to the main silica band, is observed after 1 month and 6 months of soaking, becoming increasingly evident with increasing soaking time. This indicates the dissolution of PCL over time upon immersion, whereas no conclusions can be drawn regarding the behaviour of PTHF. The small band centred around 950 cm^-1^, adjacent to the vibrational band of Si-O-C and Si-O-Si at 1070 cm^-1^, is due to the stretching of Si-OH. The larger difference in relative intensity between these two bands represents a higher degree of condensation of the Si network; a higher intensity of stretching of Si-OH means less connectivity of the silica network. After soaking, the difference is reduced, which is more evidence of degradation of the Si network.

**Figure S2.** (a) FTIR spectra for SC-250 scaffolds soaked in PBS solution for 1 month (in red solid line) and 6 months (in blue solid line) and pre-soaked sample (in dash) as the reference for comparison. Zoom-in spectra are presented in the wavenumber range of (b) 1300-700 cm^-1^, (c) 3800-2600 cm^-1^, and (d) 1800-1300 cm^-1^.

To better understand which organic components degrade upon soaking, the degradation products from the hybrid scaffolds were characterised qualitatively by ^1^H-NMR (Fig.S3). For both periods Month 0-1 and Month 3-6, the spectra for the two types of scaffold samples are comparable, suggesting that the channel size is not a significant factor influencing what organic molecules are released. Since the dissolution study was performed in PBS, the ^1^H-NMR spectrum of PBS is presented as the baseline (blue line), showing three major peaks at 1.56 (due to water present in PBS), 1.24 and 0.81 ppm. The two peaks at lower chemical shifts (𝛿) appear as residues in the SC-250 and SC-500 degradation products (blue squares) after 1 month soaking, while all three PBS peaks are visible in the sample spectra in the Month 3-6 period. Ruling out the peaks caused by PBS solution, samples in the Month 0-1 period show clear traces of PCL by comparing to the profile of PCL-COOH (red line), revealing the features around 1.4, 1.6, 2.3, 3.6 and 4.1 ppm (red stars). However, PCL features are absent in the Month 3-6 sample spectra, implying that during this period, PCL was not leached from hybrid samples or the amount was too low to be detected by ^1^H-NMR. PTHF, which dominates in the organic network of Bouncy Bioglass, normally reveals peaks at 1.55 and 3.46 ppm (areas in purple) [2]: here, despite the overlapping of the peak at 1.55 ppm with PCL-diCOOH peaks, the second feature is not obviously presenting in spectra of degraded products. Very small fluctuations around 3.46 ppm exist but are negligible as the intensity is significantly weak compared to PCL-COOH. In conclusion, the NMR results suggest that the organic part of the hybrid scaffolds degrades more in the first month than from the third to the sixth month and is mainly attributed to the dissolution of PCL-diCOOH which may come from unbonded PCL residues and degradation of the PCL molecules bonded into the hybrid network.

**Figure S3.** ^1^H-NMR spectra in CDCl_3_ of degradation products of SC-250 and SC-500 samples (black) collected from the PBS solution in two different soaking periods (Month 0-1 on the left; Month 3-6 on the right). Spectra for PCL-diCOOH (red) and PBS solution (blue) in CDCl_3_ are presented as references. Featuring peaks for PCL-diCOOH are labelled with red stars and for PBS are labelled with blue squares. Purple areas represent the chemical shifts where PTHF peaks are expected.

The weight percentages of organic parts contained in SC-250 and SC-500 hybrid samples before soaking vs. soaked for 1 month and 6 months were measured by TGA. Table S1 presents the I/O ratios, where the organic mass % is maintained at approximately 73-75% before and after soaking. The differences in the I/O ratios upon soaking are deemed not significant (less than 2%).

**Table S1.** I/O ratios measured by TGA for SC-250 and SC-500 before and after soaking in PBS for 1 month and 6 months.

| **Soaking time** | **I/O ratio measured by TGA (wt.%)** | |
| --- | --- | --- |
|  | **SC-250** | **SC-500** |
| Pre-soaking | 25.2/74.8 | 25.2/74.8 |
| 1 month | 27.0/73.0 | 25.0/75.0 |
| 6 months | 26.1/73.9 | 24.7/75.3 |

**Effects of Soaking on morphology through SEM**

The morphology of the SC-250 and SC-500 hybrid scaffolds before and after soaking was examined by SEM. The scaffold architecture is consistent with scaffolds produced previously for *in vitro* studies [3], with both scaffolds exhibiting the same strut size (~210-230 µm), since they were printed with the same nozzle size, and channel size of ~200-250 µm and ~450-500 µm for SC-250 and SC-500 respectively. Fig. S4 displays low magnification images of SC-250 and SC-500 scaffolds’ top surface, showing the grid-like architecture that is not affected by the soaking in PBS since the structure remains visible even after 6 months soaking.

The struts maintain the overall lattice shape but they are irregular and, at higher magnification, show a smooth surface with visible inherent asperities. This morphology has been commonly seen in 3D printed Bouncy Bioglass scaffolds [2] and may be attributed to the synthesis method of direct ink writing combined with the shrinkage inherent in the sol-gel process of hybrid materials [4, 5].

After 1-month of soaking, the struts for both SC-250 and SC-500 scaffolds are showing slightly rougher morphologies, with pinholes occasionally visible and randomly distributed on the struts. Small particles are occasionally observed and are deemed to be due to PBS residues.

After soaking for 6 months in PBS, the struts appeared even rougher both at low and high magnification. The latter also shows deeper penetrating pinholes. These surface features are deemed to be caused by the degradation of the material from the surface of the scaffold struts.

**Figure S4.** SEM images at low magnification of the top surface of SC-250 (left) and SC-500 (right) scaffolds with magnified images of single struts (middle, blue border for SC-250 and yellow border for SC-500). The morphology of scaffolds before soaking (top row) is compared to that of scaffolds soaked for 1 month (middle row) and 6 months (bottom row).

**Effects on Mechanical Properties**

To evaluate the effect of soaking on the mechanical behaviour of scaffolds, scaffolds with smaller channels (SC-250) were tested before soaking and after 1 and 6 months of soaking without refreshing the PBS solution (n = 5 for each analysis of each group). For each sample, the stress and strain at failure were defined at the point of failure, where the curve began to experience irregular fluctuations losing the original trend (Fig.S5a). Small steps can be noticed before the failure point in some curves, but the overall trend was kept unchanged (Fig.S5b-c). These small breakdowns were due to slight damage of the scaffolds (e.g. irregularities in a corner of the cubic sample or in a single strut) which was not considered as the failure point. Fig.S5a is an example of conventional stress-strain curves and failure points for scaffolds soaked for 1 month.

**Figure S5.** a) Stress-strain plots for SC-250 samples after soaking in PBS solution for 1 month. 5 replicates are presented in different colours with pins marked where the failure points of scaffolds during compression was determined. b) Zoom-in plot in the 0-4% strain range. c) Zoom-in plot in the 9-13% strain range.

As the scaffolds experienced barrelling under compression, the change of cross-section area needs to be counted. True strain (𝜀*) and stress (𝜎*), which can better represent the mechanical property of this material, were converted from conventional strain (𝜀_c_) and stress (𝜎_c_) at the point of failure according to Eq. 3 and Eq. 4. Results are shown in Fig. S6 and Table S2. As expected, true strains were higher than conventional strains for all groups of samples, whereas true stresses are correspondingly lower than conventional values. For the tested scaffold samples, true strains decrease from 32.2 ± 4.3 % to 28.9 ± 2.6 % then reaching 23.6 ± 2.5 % after 6 months’ soaking. True stresses show a small increase from 1.23 ± 0.08 MPa to 1.31 ± 0.24 MPa after soaking for 1 month, then decrease to 1.15 ± 0.33 MPa.

**Figure S6.** The changes in maximum true strains (ε*), conventional strains (ε_c_), true stresses (σ*), and conventional stress (σ_c_) before soaking vs. soaking time of 1 month and 6 months for SC-250 scaffolds (n = 5). Error bars represent standard deviation.

**Table S2.** A summary of true values and conventional values of stresses and strains at failure (with corresponding standard deviation) for SC-250 scaffolds, summarising the data plotted in Fig.S6 (n = 5).

|  | **Pre-soaking** | **1 Month** | **6 Months** |
| --- | --- | --- | --- |
| ε* at failure (%) | 32.2 ± 4.3 | 28.9 ± 2.6 | 23.6 ± 2.5 |
| ε_c_ at failure (%) | 27.5 ± 3.3 | 25.1 ± 2.0 | 21.0 ± 2.0 |
| σ* at failure (MPa) | 1.23 ± 0.08 | 1.31 ± 0.24 | 1.15 ± 0.33 |
| σ_c_ at failure (MPa) | 1.71 ± 0.16 | 1.74 ± 0.28 | 1.45 ±0.39 |

Before the point of failure, stress-strain curves show an elastomeric behaviour, with an increasing gradient with increasing strain. This effect is more evident at lower strain ranges, as shown in Fig. S5b-c, which are zoom-in plots of strain regions of 0-4% and 9-13%, respectively. For this reason, herein direct calculation of Young’s modulus by maximum stress and strain would not be accurate. Therefore, DMA was carried out to determine the storage modulus in four different 4% strain ranges. Fig. S7a shows an example for SC-250 samples after 1 month of soaking, where it is evident that no significant change in storage modulus (E’), loss modulus (E’’) and loss tangent (tan(𝛿)) occurred by varying the frequency (0.1, 1 and 10 Hz were tested) in the strain range of 5-9%. The same trend was observed for all samples in the four analysed strain ranges, hence we decided to report values at 1 Hz for each sample as representative values. As shown in Fig. S7a, E’ was significantly larger than E’’, consequently tan(𝛿) can be considered negligible, demonstrating the elastic behaviour of the samples in the investigated range. The storage modulus can be related to the stiffness of scaffold samples. As expected, for each time point the storage modulus increased as the strain range increased (Fig.S7b), confirming that the material becomes stiffer under higher compression. For every strain range, the storage modulus increased after soaking for 1 month, but then slightly decreased after 6 months (1-5% and 13-17%) or remained at a similar level as for 1 month (5-9% and 9-13%). Values from samples soaked for 6 months showed large error bars (data summarised in Table S3) and lead to relatively more significant change compared to other strain ranges.

During the DMA, all SC-250 samples (except one sample soaked for 6 months that broke when testing in 13-17% strain range) recovered from DMA after relaxing the compression without significant damage, evidencing the elastic behaviour of hybrids scaffolds in the investigated range.

**Figure S7.** a) Typical values of E’, E’’ and tan(δ) for SC-250 samples soaked for 1 month and tested in the 5-9% strain range at different frequencies, showing the large difference found in storage modulus (E’) and loss modulus (E’’). The loss tangent (tan(δ)) demonstrates that the contribution of viscous property is negligible. No significant variation was observed among different frequencies. b) E’ tested in 4 ranges of strain at 1 Hz before soaking and after soaking for 1 month and 6 months (n = 5). Error bars represent standard deviation.

**Table S3.** A summary of storage modulus values for SC-250 samples vs. different time of soaking, determined at 1 Hz by DMA, corresponding to Fig.S7b.

|  | **Storage modulus, E’ (MPa)** | | | |
| --- | --- | --- | --- | --- |
| **Strain range** | **1-5%** | **5-9%** | **9-13%** | **13-17%** |
| Pre-soaking | 4.0 ± 1.4 | 5.5 ± 1.9 | 6.6 ± 2.2 | 7.6 ± 2.6 |
| 1 Month | 5.2 ± 0.4 | 8.1 ± 0.9 | 9.8 ± 1.2 | 11.5 ± 2.0 |
| 6 Months | 5.0 ± 2.0 | 8.1 ± 3.1 | 9.9 ± 4.2 | 10.5 ± 4.7 |

**Supplementary Figures for “Materials and Methods” Section**

Figure S8. Photos showing the surgical steps: a) sterilisation of the operating field, b) ovine left stifle shaved and sterilised, c) incision site, d) exposed stifle joint after dislocation of the patella to visualise condyles, e) removal of the fat pad, f) punching outline of defect, g) defect outline in the condyle, h) resection of the cartilage using a dermal curette, i) final resection using an Arthrex curette, j) drilling the initial microfracture to pierce through subchondral bone, k) microfracture performed with needles to 10 mm depth, l) microfractured defects in medial and lateral condyles, m) scaffolds placed in defects, n) glue dots placed at edges of implants in defect, o) glued implants in defect, p) harvesting of the periosteal tissue from the distal femur with an osteotome, q) glueing periosteal patch to cover implants, r) final implants covered by the periosteal flap, s) deep tissue suturing the close site, t) fascia tissue suturing, u) closed operating site, v) dressing covering site, w) cotton wool wrapped for protection, x) keep in place using Vetrap.

Figure S9. QuPath images of the outlined areas of interest in yellow: a) entire defect area including regenerated tissue and void, b) regenerated tissue area, and c) neighbouring articular cartilage. The software then measures the percentage amount of positive staining in the demarked areas, here the red staining from Safranin-O (Fast Green counter stain). Scale bar 500 µm.

**References for Supplementary Information**

[1] Ting H.-K. et al., *Phosphate content affects structure and bioactivity of sol-gel silicate bioactive glasses*. Int J Appl Glass Sci, 8: 372- 382 (2017).

[2] Tallia F. et al., *Bouncing and 3D printable hybrids with self-healing properties*. Mater Horiz, 5: 849-860 (2018).

[3] Li S. et al., *Scaffold channel size influences stem cell differentiation pathway in 3-D printed silica hybrid scaffolds for cartilage regeneration*. Biomater Sci, 8: 4458-4466 (2020).

[4] Mir S.H. et al., *Review—Organic-Inorganic Hybrid Functional Materials: An Integrated Platform for Applied Technologies*. J Electrochem Soc, 165(8): B3137-B3156 (2018).

[5] Richardson B.M. et al., *Mechanobiological Interactions between Dynamic Compressive Loading and Viscoelasticity on Chondrocytes in Hydrazone Covalent Adaptable Networks for Cartilage Tissue Engineering*. Adv Healthcare Mater, 10(9): 2002030 (2021).
